# Local administration of mesenchymal stromal cells is safe and modulates the immune compartment in ulcerative proctitis

**DOI:** 10.1172/jci.insight.167402

**Published:** 2023-05-08

**Authors:** Laura F. Ouboter, Marieke C. Barnhoorn, Hein W. Verspaget, Leonie Plug, Emma S. Pool, Karoly Szuhai, Lukas J.A.C. Hawinkels, Melissa van Pel, Jaap Jan Zwaginga, Dave Roelen, Frits Koning, M. Fernanda Pascutti, Andrea E. van der Meulen – de Jong

**Affiliations:** 1 Department of Gastroenterology and Hepatology,; 2 Department of Immunology,; 3 Department of Hematology, and; 4 Department of Cell and Chemical Biology, Leiden University Medical Center, Leiden, Netherlands.; 5 Netherlands Center for the Clinical Advancement of Stem Cell & Gene Therapies (NECSTGEN), Leiden, Netherlands.; 6 Department of Internal Medicine, Leiden University Medical Center, Leiden, Netherlands.

**Keywords:** Clinical Trials, Inflammation, Inflammatory bowel disease

## Abstract

**BACKGROUND:**

Due to their immunoregulatory and tissue regenerative features, mesenchymal stromal cells (MSCs) are a promising novel tool for the management of ulcerative proctitis (UP). Here we report on a phase IIa clinical study that evaluated the impact of local MSC therapy on UP.

**METHODS:**

Thirteen refractory UP patients, with an endoscopic Mayo score (EMS) of 2 or 3, were included. Seven patients received 20–40 million allogeneic MSCs (cohort 1), while 6 patients received 40–80 million MSCs (cohort 2). Adverse events (AEs) were assessed at baseline and on weeks 2, 6, 12, and 24. Clinical, endoscopic, and biochemical parameters were assessed at baseline and on weeks 2 and 6. Furthermore, we evaluated the engraftment of MSCs, the presence of donor-specific human leukocyte antigen (HLA) antibodies (DSAs), and we determined the impact of MSC therapy on the local immune compartment.

**RESULTS:**

No serious AEs were observed. The clinical Mayo score was significantly improved on weeks 2 and 6, and the EMS was significantly improved on week 6, compared with baseline. On week 6, donor MSCs were still detectable in rectal biopsies from 4 of 9 patients and DSAs against both HLA class I and class II were found. Mass cytometry showed a reduction in activated CD8^+^ T cells and CD16^+^ monocytes and an enrichment in mononuclear phagocytes and natural killer cells in biopsies after local MSC therapy.

**CONCLUSION:**

Local administration of allogeneic MSCs is safe, tolerable, and feasible for treatment of refractory UP and shows encouraging signs of clinical efficacy and modulation of local immune responses. This sets the stage for larger clinical trials.

**TRIAL REGISTRATION:**

EU Clinical Trials Register (EudraCT, 2017-003524-75) and the Dutch Trial Register (NTR7205).

**FUNDING:**

ECCO grant 2020.

## Introduction

Nearly one-third of patients with ulcerative colitis (UC) present with disease limited to the rectum, so-called ulcerative proctitis (UP), at diagnosis (1, 2). Patients with UP suffer from many distressing symptoms, including increased stool frequency, urgency, and rectal bleeding, resulting in a poor quality of life (3). UP progresses to proximal disease extension in 37%–54% of patients and colectomy is needed in 13% of patients within 10 years of initial diagnosis (2, 4, 5). The goal of treatment in UP is mucosal healing to prevent disease progression and improve quality of life (6, 7); however, this is achieved with local therapy in only a proportion of patients (8). Nonresponders need a step-up or top-down approach, as in pancolitis (9). For these reasons, novel local therapeutic approaches without systemic side effects need to be explored.

Mesenchymal stromal cells (MSCs) have already shown great promise in various inflammatory diseases such as graft-versus-host-disease (10), sepsis (11), and Crohn’s disease–associated (CD-associated) perianal fistulas (12, 13). Furthermore, the safety of intravenous injection of MSCs has previously been investigated in UC (14, 15). Therefore, MSCs might be a good candidate to treat UP. MSCs are fibroblast-like cells with immunomodulatory and tissue regenerative features. The exact mechanism of action of MSCs is not unraveled yet, but they possess immunomodulatory properties by suppressing the proliferation of effector T cells, B cells, and natural killer (NK) cells, and by inducing the expansion of functionally suppressive regulatory T cells (16). MSCs exhibit low immunogenicity, displaying low levels of major histocompatibility complex (MHC) I and an absence of MHC II and costimulatory molecules (17, 18), and were therefore considered adequate candidates for allogeneic cell therapy, alleviating the need for the generation of autologous MSCs. Locally injected allogeneic MSCs have shown to be useful for the treatment of refractory perianal fistulas in CD (12, 13, 19) and Alofisel (Cx601, darvadstrocel) has now been approved by the European Medicine Agency for the treatment of CD-associated perianal fistulas (12, 20). Moreover, we previously showed the capability of endoscopically injected MSCs to alleviate experimental colitis in mice (21).

In this phase IIa clinical study, we evaluated the safety, tolerability, and feasibility of locally injected allogeneic bone marrow–derived MSCs in patients with refractory UP. Additionally, preliminary indicators of efficacy were obtained. We also investigated the local persistence of MSCs in the tissue after MSC therapy and the development of donor-specific human leukocyte antigen (HLA) antibodies (DSAs) following MSC injection. Moreover, changes in the composition of the immune compartment and protein biomarkers were assessed by applying single-cell mass cytometric and proteomic analysis of rectal biopsies. Altogether, this comprehensive study was found to set the stage for larger, continual studies to further explore the clinical application of local MSC therapy in UP.

## Results

### Study population

**Figure 1:**
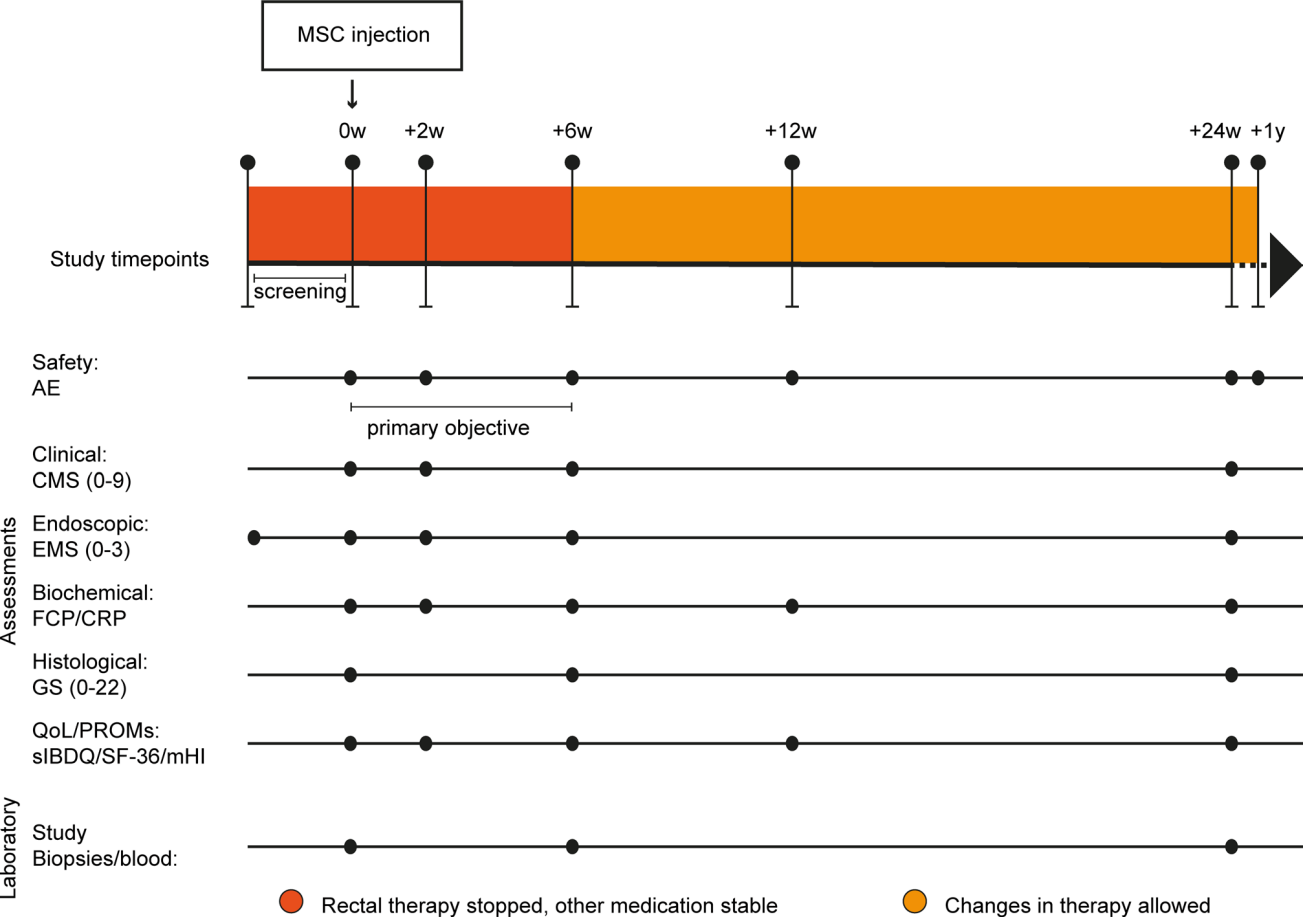
Study design. Time points are indicated in weeks (w) or years (y). AE, adverse events; CMS, clinical Mayo score; EMS, endoscopic Mayo score; FCP, fecal calprotectin; CRP, C-reactive protein; GS, Geboes score; QoL, Quality of Life; PROMs, patient-reported outcome measures; sIBDQ, Short Inflammatory Bowel Disease Questionnaire; SF-36, Short Form-36; mHI, mobile Health Index (56).

In total, 24 patients were evaluated for their eligibility. To be eligible for the study, patients must have had inflammation limited to the rectum (endoscopic Mayo score [EMS] 2 or 3) that did not respond to conventional medical therapy (rectal mesalamine and rectal corticosteroid therapy). Eleven patients were excluded because of dysplasia in the rectum, disease not limited to the rectum, or EMS of 1 or lower. Finally, 13 patients with moderate to severe and refractory UP were included in the study (according to the study design in Figure 1), of whom 10 were female (76.9%). The patients had a median age of 48 (range, 31.0–57.7) years. Due to the COVID-19 pandemic, inclusion ceased early and the original aim of 14 patients was not met. The first patient received intervention in February 2019 and the last patient in March 2020. All patients completed the study up to week 24. There was a long-term follow-up, with a checkup at 1 year after MSC treatment through a telephone call. Additional baseline characteristics of the patients are summarized in Table 1.

### Safety, tolerability, and feasibility

There were no serious adverse events reported in the first 6 weeks after MSC therapy. The most commonly reported adverse events in the first 6 weeks included abdominal pain, abdominal cramps, nausea, vomiting, and flatulence (Supplemental Table 1; supplemental material available online with this article; https://doi.org/10.1172/jci.insight.167402DS1). Two patients experienced fever in the first days after MSC injection. There were only mild to moderate adverse events reported up to week 24 and week 52. In 1 patient, a relative stenosis, a so-called lumen with scars, over a length of 6 cm was observed in the rectum on week 12 and persisted until week 24. The endoscope could pass. This area was severely inflamed at baseline. A large ulcus and a relative stenosis were observed on week 12, but a healing tendency was present. Biopsies were analyzed and only inflammation was reported. One year after MSC injection, the stenosis was no longer observed, but a small ulcus without worrisome features was noticed. Given the mild to moderate adverse events, no significant changes in vital signs, and the global judgment of the investigator, it was concluded that the therapy was tolerable. Regarding the feasibility, there were generally no practical problems and complaints encountered in the study’s execution and no dropouts in the study. No differences in safety, tolerability, and feasibility were found between the 2 cohorts receiving different dosing of MSCs. For this reason and the low sample size, cohorts 1 and 2 were taken together for the evaluation of the clinical efficacy.

### Efficacy

#### Clinical and endoscopic results.

**Figure 2:**
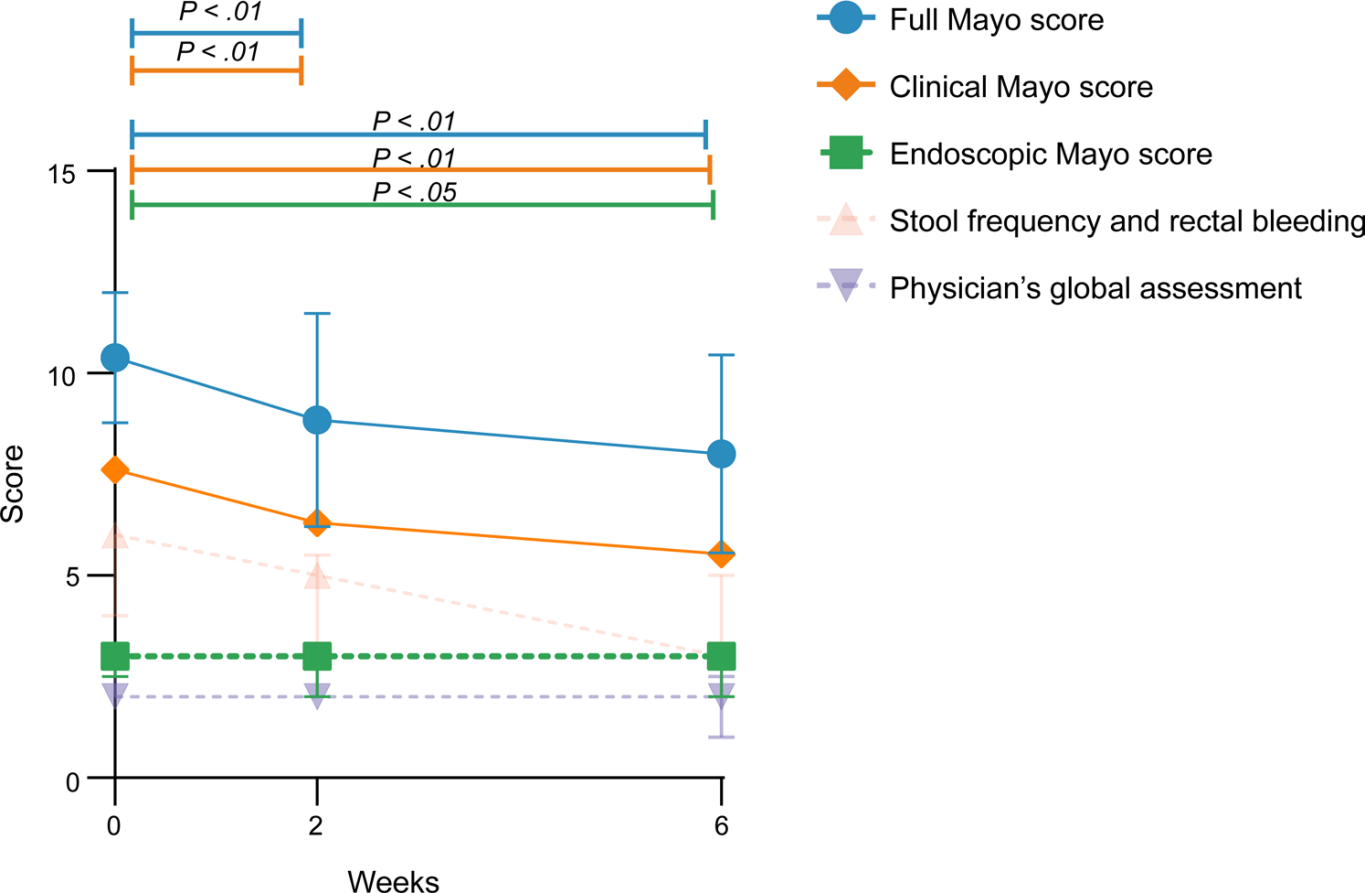
Mean full Mayo score with corresponding subscores at baseline and on weeks 2 and 6. Data are presented as mean ± SD. Wilcoxon’s signed-rank test was performed.

The full Mayo score (FMS) and the clinical Mayo score (CMS) significantly decreased on weeks 2 and 6, compared with baseline. The EMS significantly improved on week 6, compared with baseline (Figure 2).

Clinical response (FMS) was observed in 1 patient on week 2 (7.7%), and in 4 patients (30.8%) on week 6. Clinical remission was achieved in 1 patient (7.7%) on week 2, and in none of the patients on week 6. Endoscopic response was observed in 3 patients (23.1%) on week 2, and in 4 patients (30.8%) on week 6. No deterioration was observed at any time point (Supplemental Table 2). In total, 6 of 13 patients (46.2%) started, switched, or intensified the dose of treatment within 12 weeks of follow-up and an additional 6 patients in the next 12 weeks. In 4 patients, this was just rectal therapy and in 1 patient rectal therapy in combination with oral 5-aminosalicylic acid (5-ASA) (Supplemental Table 3). Remarkably, 2 of these patients showed clinical remission and 3 patients showed clinical response on week 24 compared with baseline, whereas before MSC injection these patients were refractory to these treatments. Although no conclusions can be drawn regarding efficacy beyond week 6, results are shown in Supplemental Table 2. The mean FMS with corresponding subscores at baseline and follow-up visits on weeks 2, 6, and 24 are shown in Supplemental Figure 1.

#### Fecal calprotectin and C-reactive protein.

In total, 9 patients (69.2%) had a decrease in their fecal calprotectin (FCP) 2 weeks after MSC injection, and 6 patients (46.2%) after 6 weeks. Just 1 patient showed normalization of FCP on week 6. No changes of clinical interest were observed within the C-reactive protein (CRP) values (Supplemental Table 4).

#### Histologic activity.

On week 6, 2 patients (15.4%) showed histological response (Geboes score [GS] ≤ 12), 5 (38.5%) showed histological remission (GS ≤ 6), and 6 (46.2%) showed no improvement, compared with baseline (Supplemental Table 5).

#### “Quality of life” and “Patient-reported outcome measures” questionnaires.

No significant improvement was measured in the Short Inflammatory Bowel Disease Questionnaire, Short Form-36, and mobile Health Index on week 2. However, there was significant improvement on week 6, compared with baseline (Supplemental Table 6).

### Local persistence of MSCs after 6 weeks

**Figure 3:**
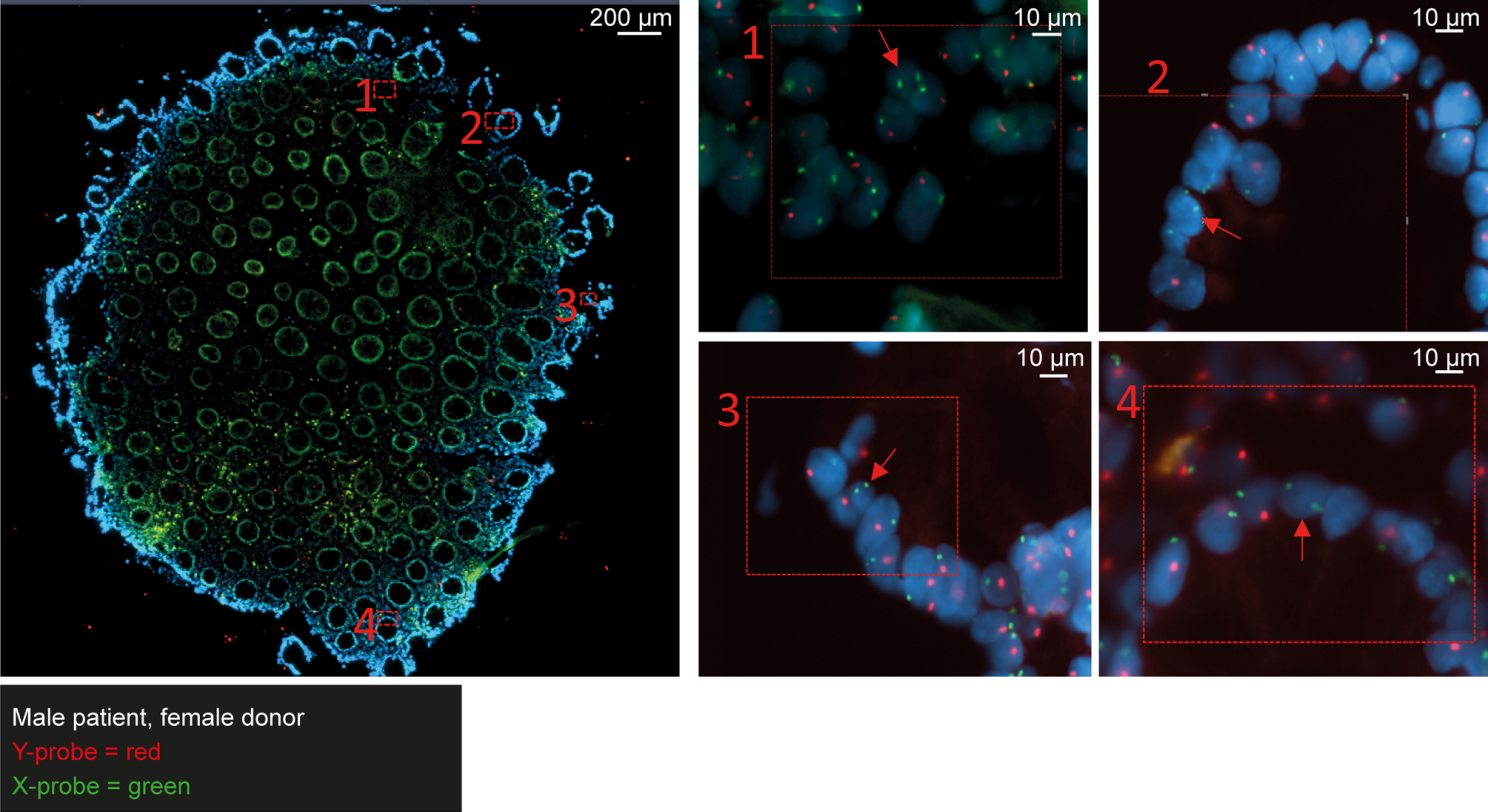
MSCs persist in the rectal mucosa after 6 weeks. A representative image is displayed of a rectal biopsy from a male patient that received allogeneic MSCs from a female donor. In multiple regions, 2 X chromosomes (in green) were detected in close proximity in the nucleus of a cell (red arrow). Probes detect the presence of chromosome Y (in red) and chromosome X (in green). DAPI (4′-6′-diamidino-2-phenylindole, in blue) was used to stain the DNA. Scale bars: 200 μm (left) and 10 μm (right).

FISH, based on X and Y chromosome–specific probes, was performed on biopsies collected 6 weeks after MSC injection to investigate whether MSCs locally persisted in the tissue mucosa. This technique relies on sex differences between patient and MSC donor; for that reason, biopsies from 10 of 13 patients were analyzed, 1 of whom was excluded due to technical problems. Eventually, in 4 of 9 analyzed patients, 2 chromosomes of the opposite sex were discovered in the rectal biopsies, indicating long-term persistence of the locally applied MSC (Figure 3). Both responders and nonresponders were identified among the patients that showed local persistence of MSCs after week 6 (Supplemental Table 7).

### Local allogeneic MSC therapy induces the generation of both HLA class I and class II DSAs

Despite the low immunogenicity of MSCs, as described in the literature, the use of allogeneic MSCs may induce immune responses triggered by HLA mismatches between donor and recipient. Therefore, blood samples from all 13 MSC-treated patients were analyzed for the presence of DSAs at baseline and on weeks 6 and 24. On week 6, newly induced HLA class I–specific DSAs were detected in 4 patients (patients 4, 6, 9, and 11), while in 1 of these patients (patient 9) newly induced HLA class II DSAs were also found. Here, patient 11 was already presensitized as indicated by the presence of HLA class I and class II at baseline, which were boosted by MSC therapy on week 6, with sustained high levels through week 24. This patient also developed new HLA class I DSAs, which were still detectable on week 24. In the other 3 patients (patients 4, 6, and 9) the newly formed HLA class I and class II DSAs were no longer detectable on week 24 (Supplemental Tables 8 and 9). Both responders and nonresponders were identified among the patients that developed DSAs (Supplemental Table 7). In order to determine whether these DSAs were potentially able to kill the locally injected donor MSCs, a complement-dependent cytotoxicity (CDC) assay was performed. CDC panel screening of serum from patients 4, 6, and 9 was negative, indicating that the DSAs in these patients were not complement activating. In patient 11, CDC reactivity was observed, caused by antibodies directed against HLA-A2, HLA-DR4, HLA-DQ8, and HLA-DQ3 (Supplemental Table 10).

### Local MSC therapy is associated with modulation of proinflammatory proteins in rectal biopsies

**Figure 4:**
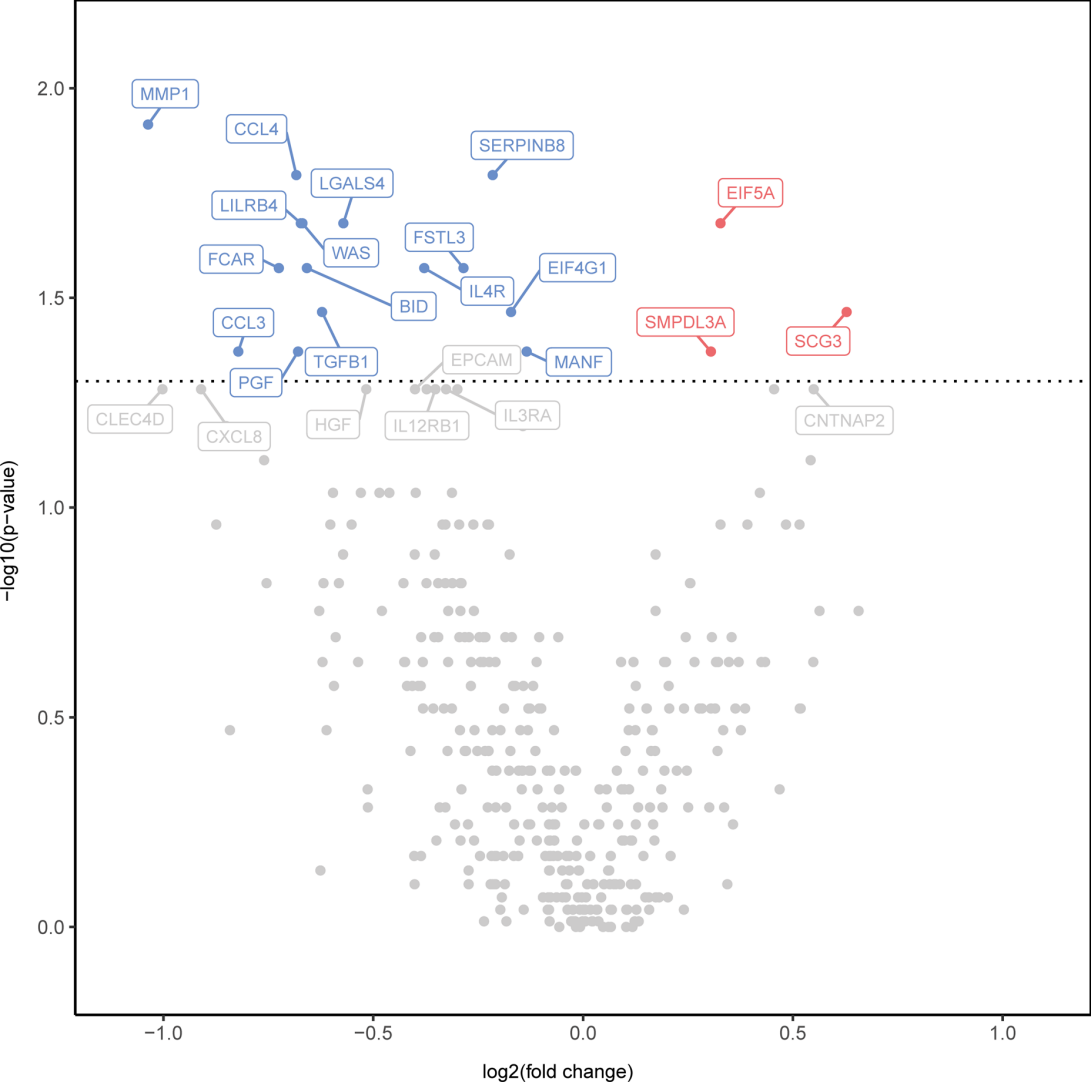
Local MSC therapy is associated with modulation of the inflammatory proteins in rectal biopsies. A volcano plot showing the statistical significance and fold change in protein levels 6 weeks after MSC injection compared with baseline. Decreased levels of proteins are indicated in blue and increased levels of proteins in red. Gray points indicate no significant change. Wilcoxon’s signed-rank test was performed.

We applied the Olink Explore 384 Inflammation panel to evaluate the presence and abundance of inflammatory proteins in rectum biopsies before and after MSC injection. A volcano plot visualization of the results indicates that the levels of 3 proteins were higher 6 weeks after MSC injection, while 15 proteins were found to be lower (Figure 4 and Supplemental Figure 1). Proteins with increased levels were related to mRNA turnover (EIF5A) (22), angiogenesis (SCG3) (23), and nucleotide triphosphate breakdown (SMPDL3A) (24), while proteins showing decreased levels were related to immune cell recruitment (CCL3 and CCL4) (25), tissue remodeling (MMP1) (26), IgA-mediated effector responses (FCAR) (27), regulation of inflammation (TGF-β1, FSTL3, and LILRB4) (28–30), epithelial cell adhesion (SERPINB8 and LGALS4) (31, 32), and apoptosis (BID) (33), among others, pointing toward decreased inflammation and potential epithelial proliferation.

### Reduction in activated CD8+ T cells and CD16+ monocytes and an increase in CD127+ monocytes/macrophages and tissue-resident NK cells after local MSC therapy

**Figure 5:**
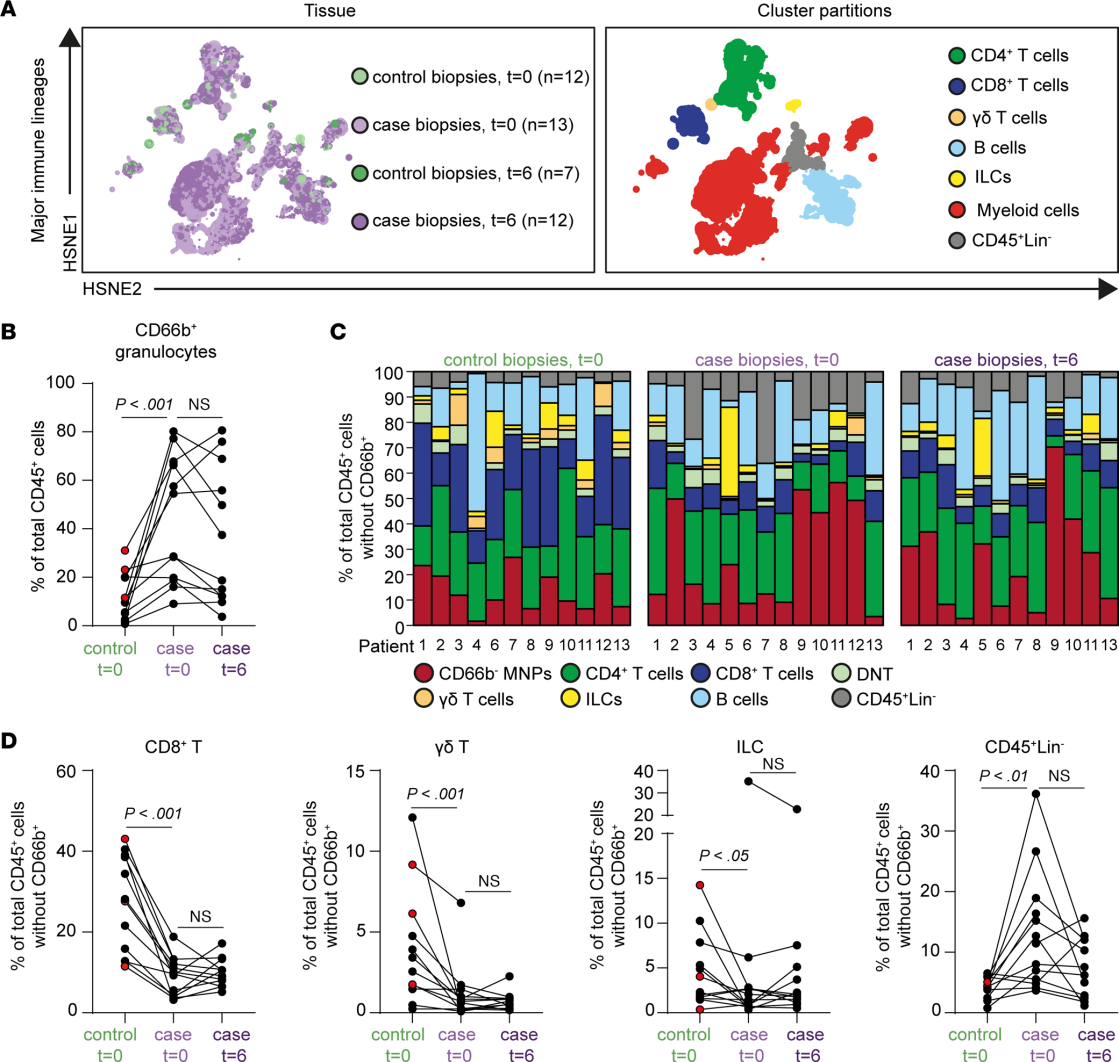
CD8^+^ T cells, γδ T cells and, innate lymphoid cells are decreased in case biopsies compared with control biopsies but do not change significantly upon local MSC injection. (**A**) HSNE embedding showing 1.6 × 10^4^ landmarks representing immune cells (3.4 × 10^6^ cells) isolated from control biopsies at baseline (*n* = 12) and on week 6 (*n* = 7) and from case biopsies at baseline (*n* = 13) and on week 6 (*n* = 12). Colors represent the biopsies at different time points (left) and the other immune lineage clusters (right). (**B**) Frequencies of CD66b^+^ granulocyte cluster in control biopsies at baseline (*n* = 12), case biopsies at baseline (*n* = 13), and after 6 weeks (*n* = 12) as a percentage of total CD45^+^ cells. (**C**) Composition of the major immune lineages, CD66b^+^ granulocytes excluded, in individual patients (*n* = 13) in control biopsies (left), case biopsies (middle) at baseline, and case biopsies on week 6 (right), represented as vertical bars. The size of the colored segments represents the proportion of the cells as a percentage of total CD45^+^ cells in the samples. Colors are as in **A**. (**D**) Frequencies of the CD8^+^ T cells, γδ T cells, ILCs, and CD45^+^Lineage^–^ cells from individual samples (*n* = 12) (control biopsies and case biopsies at baseline and case biopsies on week 6). Each dot represents an individual sample. Red dots are biopsies with mild endoscopic inflammation. NS, not significant. Wilcoxon’s signed-rank test was performed.

Changes in the mucosal immune cell composition upon local MSC injection were explored using high-dimensional mass cytometry. Single-cell suspensions prepared from freshly obtained biopsies were stained with a 41-antibody panel (Supplemental Table 10) followed by data acquisition on a Helios mass cytometer. Next, a dimensionality reduction analysis (e.g., hierarchical stochastic neighbor embedding [HSNE]) was performed on all acquired CD45^+^ cells (3.4 million cells in total) isolated from case, all inflamed, and control, mostly noninflamed, biopsies. This identified the presence of CD4^+^ T cells, CD8^+^ T cells including double-negative (DN) T cells, TCRγδ T cells, innate lymphoid cells (ILCs), B cells, myeloid cells (CD66b^+^ granulocytes and CD66b^–^mononuclear phagocytes [MNPs]), and CD45^+^Lineage^–^ cells in all biopsies (Figure 5A). CD66b^+^ granulocytes were significantly enriched in case biopsies compared with control biopsies at baseline (*P* = 0.001), ranging from 10 % to up to 80% of all CD45^+^ cells. Their abundance, however, was not significantly different in the case biopsies 6 weeks after MSC therapy (Figure 5B).

Given the large variation in granulocyte content in the case biopsies, granulocytes were removed from the total CD45^+^ cells for the comparison of other major lineages (Figure 5C). This analysis revealed that the percentages of CD8^+^ T cells, γδ T cells, and ILCs were significantly lower (*P* = 0.0005, *P* = 0.0010, *P* = 0.0034, respectively; Figure 4D) in case biopsies compared with control biopsies at baseline, whereas the percentage of CD45^+^Lin^–^ cells was increased (*P* = 0.005; Figure 5D). In contrast, the CD66b^–^ MNPs, CD4^+^ T cells, DN T cells, and B cells did not differ significantly between case and control biopsies at baseline (Supplemental Figure 2A). MSC therapy did not lead to significant changes in the frequencies of the major immune lineages in the case biopsies on week 6 compared with baseline (Figure 4D and Supplemental Figure 2A).

**Figure 6:**
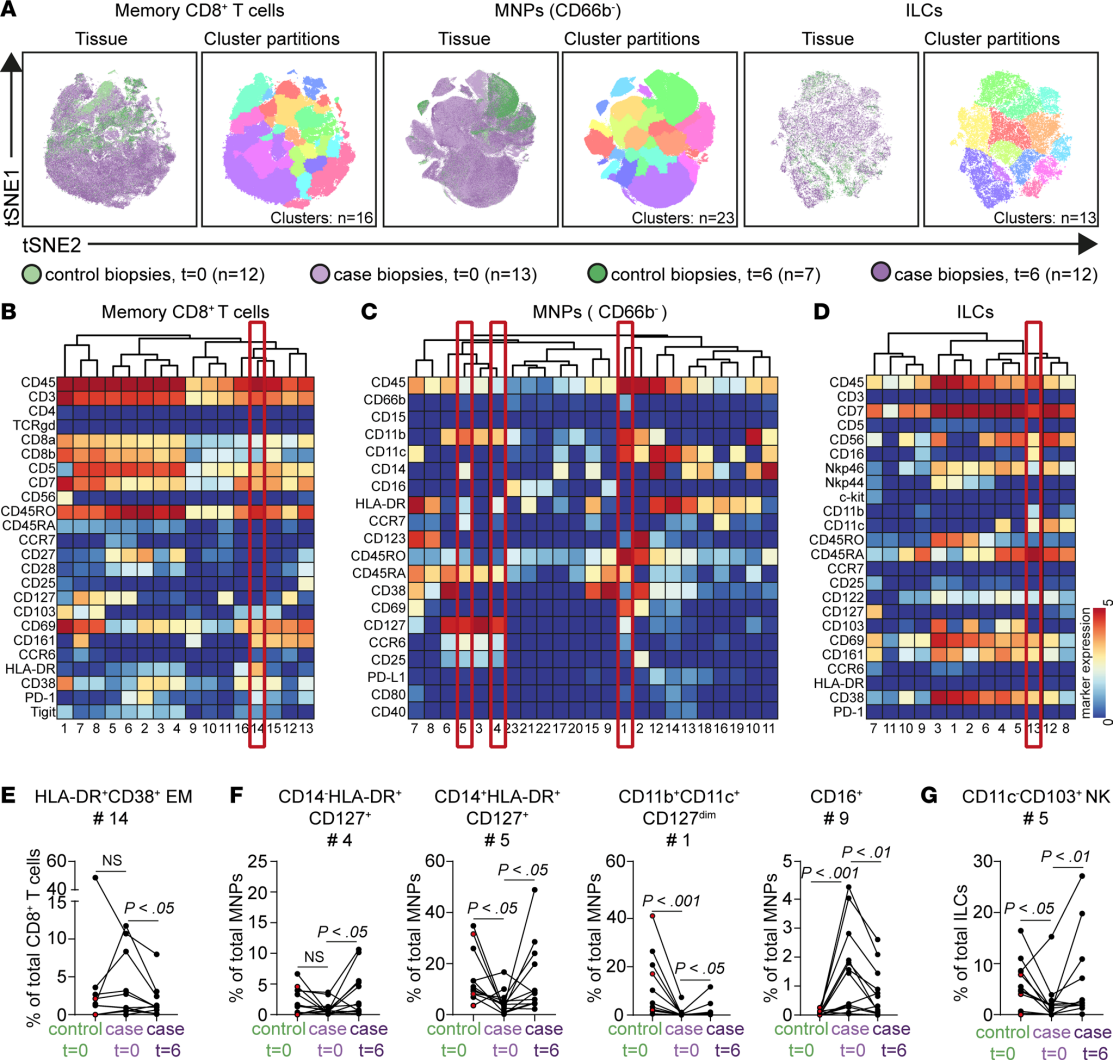
Reduction in activated CD8^+^ T cells and CD16^+^ monocytes and increased CD127^+^ monocytes/macrophages and tissue-resident NK cells after local MSC therapy. (**A**) t-SNE embedding of memory CD8^+^ T cell compartment (in total 1.7 × 10^5^ cells), CD66b^–^ mononuclear phagocyte (MNP) compartment (in total 4.2 × 10^5^ cells), and ILC compartment (in total 4.6 × 10^4^ cells). Colors represent the biopsies at different time points (left) and the different clusters (right). (**B**–**D**) Heatmaps of the memory CD8^+^ T cell compartment (**B**), MNP (CD66b^–^) compartment (**C**), and ILC compartment (**D**) showing median marker expression values (top). (**E**–**G**) Frequencies of selected clusters within the CD8^+^ T cell compartment (**E**), MNP compartment (**F**), and ILC compartment (**G**) in biopsies from control biopsies at baseline (*n* = 12), case biopsies at baseline (*n* = 12), and after 6 weeks (*n* = 12) as a percentage of the corresponding major lineages. Each dot represents an individual sample. Red dots are biopsies with mild endoscopic inflammation. NS, not significant. Wilcoxon’s signed-rank test was performed.

We further investigated the major immune lineages separately at the single-cell level using t-distributed stochastic neighbor embedding (t-SNE) analysis to achieve maximal resolution within the data set. Examples of the embedding of the memory CD8^+^ T cells, CD66b^–^ MNPs, and ILCs are shown in Figure 6A.

This analysis identified 16 phenotypically distinct immune cell clusters within the memory CD8^+^ T cells. Here, a cluster of HLA-DR^+^CD38^+^CD161^+^ effector memory CD8^+^ T cells (cluster 14) was significantly decreased in the case biopsies 6 weeks after local MSC injection compared with the case biopsies at baseline (*P* = 0.04; Figure 6, B and E). This cluster was further characterized by the coexpression of CD69, and dim expression of PD-1, pointing to tissue residency and cellular activation, respectively.

The t-SNE analysis of the CD66b^–^ MNPs revealed 23 distinct immune cell clusters. Strikingly, we found that 4 of these MNP cell clusters (clusters 3–6) expressed CD127 (IL-7Ra subunit), generally considered a lymphoid lineage marker. Two of these clusters (clusters 4 and 5) were significantly increased 6 weeks after MSC injection compared with baseline (both *P* = 0.02; Figure 6, C and F) in the case biopsies. These clusters displayed differential expression of CD14 (absent in cluster 4, low for cluster 5), HLA-DR (clusters 4 and 5), and CCR7 (absent in cluster 4, low for cluster 5). Moreover, a CD11b^+^CD11c^+^CD69^+^CD127^dim^ cell cluster was similarly increased 6 weeks after MSC therapy (*P* = 0.03; Figure 6, C and F) in the case biopsies. Conversely, the frequency of CD16^+^ nonclassical monocytes (cluster 9) decreased (*P* = 0.009; Figure 6, C and F).

Finally, in-depth profiling of the ILC compartment revealed 13 distinct immune cell clusters, of which 1 NK cell cluster (cluster 5), expressing the tissue residency markers CD69 and CD103, was increased in frequency in the case biopsies 6 weeks after local MSC therapy (Figure 6, D and G).

Strikingly, 6 weeks after MSC therapy, the frequencies of most of these populations resembled more closely the control regions within the same patient (MNP: clusters 5, 1, and 9; ILC: cluster 5; Figure 6, C, D, F, and G).

Altogether, these data demonstrate that local MSC injection is associated with a reduction in activated CD8^+^ T cells and CD16^+^ monocytes, and an enrichment in MNPs expressing CD127^+^ and tissue-resident NK cells in the inflamed rectum in the case biopsies.

## Discussion

To our knowledge, this is the first clinical study in which allogeneic MSCs were locally injected into the inflamed rectal mucosa of patients with refractory UP. We show that local administration of allogeneic MSCs was safe, feasible, and well tolerated in patients with refractory UP. Reported adverse events through 1-year follow-up were all mild to moderate in severity and most generally considered related to inflammatory bowel disease (IBD). This is in agreement with clinical trials showing that local injection of allogeneic MSCs are a safe treatment for perianal fistulas (12, 13, 34–36).

Although this study was not designed and powered to determine efficacy, local MSC therapy for UP showed encouraging signs of clinical efficacy. The FMS improved significantly 2 and 6 weeks after MSC injection. Of note, all rectal therapy was withdrawn at least 2 weeks before MSC injection, which could have triggered an exacerbation. Twelve of the 13 patients required start or switch of therapy between week 6 and up to week 52. Four of these 12 patients required only rectal therapy, 2 of whom achieved clinical remission on week 24. Interestingly, 1 patient achieved endoscopic, biochemical, and histological remission on week 24, with only the reintroduction of rectal mesalazine and rectal steroids, suggesting the ability of local MSC therapy to make a patient more sensitive to previous medication. In addition, the endoscopic, biochemical, and histological parameters improved in a proportion of patients, with the greatest improvement for all parameters on week 24.

Based on our previous clinical trial for fistulizing CD (13), we assigned the first 7 patients to a cohort in which 5 × 10^6^ cells were injected at 4–8 spots and the next 6 patients to a cohort in which 10 × 10^6^ cells at 4–8 spots were injected. No differences in safety were observed between the 2 cohorts. Larger phase III trials are needed to confirm which dose would be best regarding clinical efficacy.

In our study, we used allogeneic bone marrow–derived MSCs from healthy donors. A major advantage of an allogeneic product is the possibility of its use as an “off-the-shelf” product, avoiding the need for delays due to autologous cell expansion before treatment. A disadvantage of autologous MSCs is their risk of contributing to disease development; they may be affected by the underlying disease, as observed in multiple myeloma and systemic sclerosis (18, 37). The promising results of local allogeneic MSCs in CD-associated perianal fistulas strengthen our choice for allogeneic instead of autologous MSCs (13). On the other hand, allogeneic MSCs were found to induce an immune response in 4 patients, as evidenced by the presence of newly induced HLA class I–specific antibodies, and newly induced HLA class II–specific antibodies in 1 patient. Moreover, MSC therapy boosted preexisting HLA class I and HLA class II antibodies in 1 patient, with sustained high levels on week 24. This would advocate that in the future, patients with a history of alloantigen exposure should be screened for preexisting HLA antibodies. Assessment of the peripheral donor-specific memory B cell pool may also be clinically desirable to obtain good insight into the potential humoral alloimmune response in MSC recipients with a history of pregnancy, blood transfusion, or transplantation (38). Our results are partly in line with those of Avivar-Valderas and colleagues who detected newly formed DSAs against HLA class I or preexisting HLA class I DSAs that were boosted in presensitized patients treated with local MSC therapy (17). Interestingly, to the best of our knowledge HLA class II DSAs in response to MSC therapy have not been described previously. Thus, despite the fact that MSCs were for a long time considered to be poorly immunogenic (39, 40), they are able to induce alloantibody responses. Nevertheless, in a 4-year follow-up study performed by our group on local allogeneic bone marrow–derived MSC therapy in perianal fistulas, no DSAs were found (19). In recent studies, we and others observed that MSCs are able to express HLA class II, particularly after contact with proinflammatory cytokines (39, 41–43). Thus, the proinflammatory environment (e.g., IFN-γ) in the inflamed intestine may induce the upregulation of HLA class II on MSCs, resulting in HLA class II–specific antibody formation. Such antibodies may hamper reinjection of MSCs, as this could cause a loss of efficacy of the MSCs.

This clinical study offered a great opportunity to study some important working mechanism of MSCs in the tissue, as we had unique access to pre- and post-MSC–treated biopsies. We were able to illustrate the persistence of MSCs in the tissue and the potential immunomodulatory properties of locally applied MSCs. Previous studies showed that when MSCs are administered intravenously, many are trapped in the lungs (41, 44), undergo apoptosis, and are phagocytosed by macrophages that subsequently acquire an antiinflammatory phenotype (11, 44, 45). When locally administered, MSCs would be able to exert their immunosuppressive function and epithelial regenerative capacities by either direct cell-to-cell contact or through the local production of exosomes (46, 47), chemokines, and cytokines (48). Our group previously showed that MSCs could be detected up to 6 days after local injection in mice with experimental colitis (21). Strikingly, in the current clinical trial, we observed that locally administered MSCs persisted in the tissue for up to 6 weeks in at least 4 patients. It would be interesting to correlate the persistence of MSCs with clinical outcomes and the presence of DSAs in subsequent larger studies.

We determined potential immunomodulatory properties and applied high-dimensional mass cytometric analysis to perform extensive phenotypic analysis of the innate and adaptive immune compartment in the tissue biopsies. CD8^+^ effector memory T cells are associated with inflammation in IBD (49) and trigger tissue destruction and produce TNF-α in patients with UC (50). Interestingly, in our UP cohort, activated HLA-DR^+^CD38^+^ CD8^+^ T cells decreased upon local MSC injection. Moreover, we observed an increase in tissue-resident CD103^+^ NK cells, which could point to epithelial repair (51). Strikingly, we observed the presence of CD127^+^ MNPs in the intestinal biopsies, while CD127^+^ is generally considered a lymphoid marker. Zhang and colleagues, however, identified a CD127^+^ monocyte population nearly absent in healthy individuals but abundantly present in rheumatoid arthritis that has antiinflammatory properties (52). In our analysis, we observed a higher abundance of these CD127^+^ MNPs in control biopsies compared with case biopsies. Interestingly, this population increased in the case biopsies that were treated with MSCs. These results warrant further exploration of the role of these innate and adaptive immune cell subsets in UC and the role of MSC application in their regulation.

In conclusion, the results from our clinical phase IIa study revealed that local administration of allogeneic bone marrow–derived MSCs was safe, feasible, and well tolerated in patients with UP, also in the long term. Local MSC therapy showed encouraging signs of clinical efficacy and may have clinical benefits in patients with refractory UP. Moreover, significant changes in the composition of the tissue-resident immune compartment were observed upon local MSC application. Together, this sets the stage for a larger, double-blind, placebo-controlled clinical trial to further explore at least single local MSC therapy as a potential treatment for IBD and exactly specify their immunoregulatory properties at the mucosal level.

## Methods

### Study design

We performed a prospective phase IIa study in 13 adult patients with refractory UP from July 2018 through March 2021 at the Leiden University Medical Center, the Netherlands. Patients received locally injected MSCs during endoscopy in the inflamed rectum at baseline and had follow-up visits on weeks 2, 6, 12, 24, and 52. Patients in the first cohort (*n* = 7) received 20 or 40 × 10^6^ allogeneic MSCs (depending on the total length of inflammation: ≤7 cm, 20 × 10^6^; >7 cm, 40 × 10^6^), while patients in the second cohort (*n* = 6) received 40 or 80 × 10^6^ allogeneic MSCs. Safety data of the first cohort were assessed by an independent data monitoring committee before including patients in the second cohort. The isolation, expansion, and preparation of MSCs for injections are described in the Supplemental Methods. This study was officially registered for the Netherlands Trial Register under the EudraCT number 2017-003524-75, but can currently be found in the EU Clinical Trials Register.

#### Patient selection.

We enrolled patients 18 years or older with refractory UP (failed on both rectal 5-ASA and rectal corticosteroid therapy after at least 4 weeks of therapy) with an EMS of 2 or 3. Mild inflammation in other parts of the colon was accepted with a maximum EMS of 1. A stable dose of drugs used at baseline was mandatory (5-ASA ≥4 weeks; oral corticosteroids ≥2 weeks and ≥2 months for other medications with at least 3 months of use). All rectal therapy was stopped 2 weeks before endoscopic injection of MSCs. Therapy at baseline remained stable during the first 6 weeks of MSC therapy. After week 6, any medication could be changed or started at the discretion of the treating physician. The full inclusion and exclusion criteria are reported in the Supplemental Methods.

#### Endoscopic administration of MSCs.

First, the length of inflammation was measured. If the total length of inflammation was 7 cm or less, the MSCs were injected submucosally in the middle of the inflamed rectum in 4 quadrants (4 spots total). If the inflammation exceeded 7 cm, the MSCs were injected at one-quarter and three-quarters of the inflamed rectum in all 4 quadrants (8 spots total). At each injection spot, first a submucosal blister was created by the injection of saline. Subsequently, the MSCs were injected into this blister and the needle was rinsed with saline, while still in the blister. A total of 5 × 10^6^ MSCs (in the first cohort, *n* = 7) or 10 × 10^6^ MSCs (in the second cohort, *n* = 6) were injected in each spot.

### Assessments

#### Safety, feasibility, and tolerability.

Patients were monitored for adverse events during MSC administration and at each study visit according to toxicity and complications based on Common Terminology Criteria for Adverse Events criteria (v4.0). Local MSC therapy in patients with UP was safe when no serious adverse events were reported up to 6 weeks after administration. Evaluation of tolerability was based on adverse event monitoring, measurements of vital signs, as well as the investigators’ global judgment of tolerability. The patient was not explicitly asked whether the therapy was well tolerated. Evaluation of feasibility was based on practical problems and complaints that were encountered in the execution of the study. The therapy was found feasible when there were less than 20% practical dropouts.

#### Efficacy.

This study was not designed to investigate the clinical efficacy of local MSC therapy, so it was evaluated only in an exploratory context. The FMS (score 0–12), the CMS (score 0–9), and the EMS (score 0–3) were evaluated at all time points. Clinical response on weeks 2, 6, and 24 was defined as a decrease in the FMS from baseline of 30% or greater and 3 or more points, along with either a decrease from baseline in rectal bleeding subscore of 1 or greater or rectal bleeding subscore of 1 or lower (53). Clinical remission was achieved at an FMS of 2 or higher (54), without an individual subscore of greater than 1. The EMS at baseline and on weeks 2 and 6 was evaluated in retrospect based on good quality pictures, blindly by 2 experienced gastroenterologists. The gastroenterologists had no knowledge of the patients and time points corresponding to the pictures. The scores of the 2 gastroenterologists matched across all pictures. The EMS was also evaluated prospectively by the nonblinded endoscopist and described in the endoscopy report. In a single patient at 1 time point, there was a discrepancy in the prospective score of the endoscopist and the score of the blinded gastroenterologists. The score of the blinded gastroenterologists was chosen. Endoscopic response and remission on weeks 2, 6, and 24 were defined as a decrease of 1 or greater in EMS compared with baseline and an EMS of 0, respectively. The inflammatory markers CRP and FCP were measured at baseline and on weeks 2, 6, 12, and 24. For histological assessment, rectal biopsies were taken from MSC-treated regions at baseline and after 6 and 24 weeks. The rectal biopsies were subject to central reading by 2 independent qualified gastrointestinal histopathologists who were blinded to clinical outcome data, patient identification, and biopsy collection time points. Disagreements between the pathologists were resolved by re-reviewing the histological score, after which consensus was achieved. Histological response was defined as a GS of 12 or lower and histological remission was defined as a GS of 6 or lower (55).

### Human samples

From the included patients (*n* = 13), additional biopsies were collected for Olink proteomics and mass cytometry, next to a biopsy for histopathology which was also used for FISH (Figure 1) analysis. For mass cytometry, 2 case and 2 control biopsies were collected at baseline and on week 6 from each patient. The case biopsies were obtained from an inflamed area in the rectum where the MSCs were injected at baseline. The control biopsies were obtained from the sigmoid (at baseline: EMS 0 in 9 patients, EMS 1 in 2 patients) and from the rectum (EMS 1) in 1 patient with an ileorectal anastomosis (IRA). No control biopsies were collected from 1 patient with an IRA because the whole rectum showed moderate to severe inflammation. Eventually, 5 control biopsies on week 6 and 1 case biopsy on week 6 were excluded for further analysis due to a low number of total CD45^+^ cells (<1000) or poor quality of the sample.

### Laboratory methods for supportive research

This clinical study allowed us to study some important mechanisms of action of MSCs in humans. Sera of patients were screened for DSAs at baseline and on weeks 6 and 24 using Luminex single-antigen bead assays. When DSAs were detected, CDC was assessed. FISH, based on X and Y chromosome–specific probes, was performed on biopsies collected 6 weeks after MSC injection to investigate whether MSCs locally persisted in the tissue mucosa. The immunomodulatory effects of local MSC therapy were evaluated by mass cytometry and proteomics in biopsies at baseline and on week 6. Further details are described in the Supplemental Methods.

### Statistics

Data were analyzed using SPSS (v25), GraphPad Prism (v9.3.1), and R (Olink Analyze package v3.1.0 in R v4.0.5). Analyses included the Mann-Whitney *U* test and Wilcoxon’s signed-rank test for paired data. *P* values less than 0.05 were considered significant. Due to the exploratory nature of this study and low sample size, correction for multiple testing was not performed.

### Study approval

This clinical trial is registered at https://clinicaltrialsregister.eu (EudraCT 2017-003524-75) and Dutch Trial Register (NTR7205). The study was approved by the Medical Ethical Committee of the Leiden University Medical Center (LUMC) (protocol NL63157.000.17) and the Central Committee on Research involving Human Subjects (The Hague, the Netherlands). All patients gave written informed consent. All authors had access to the study data and reviewed and approved the final manuscript.

## Author contributions

MCB, HWV, MVP, JJZ, and AEMJ contributed to the study design. LFO, MCB, LP, KS, and DR generated data. LFO, MCB, ESP, LJACH, DR, FK, MFP, and AEMJ analyzed and interpreted the data. LFO wrote the first draft of the manuscript. LFO, MCB, HWV, LP, ESP, KS, LJACH, MVP, JZ, DR, FK, MFP, and AEMJ revised the manuscript. LFO and MCB are co–first authors. The order of their names was determined by the fact that LFO acquired most of the data, performed much of the experimental work and data interpretation, and MCB had a major contribution in designing the study. All authors approved the final version of the manuscript.

## Supplementary Material

Supplemental data

## Figures and Tables

**Table 1:**
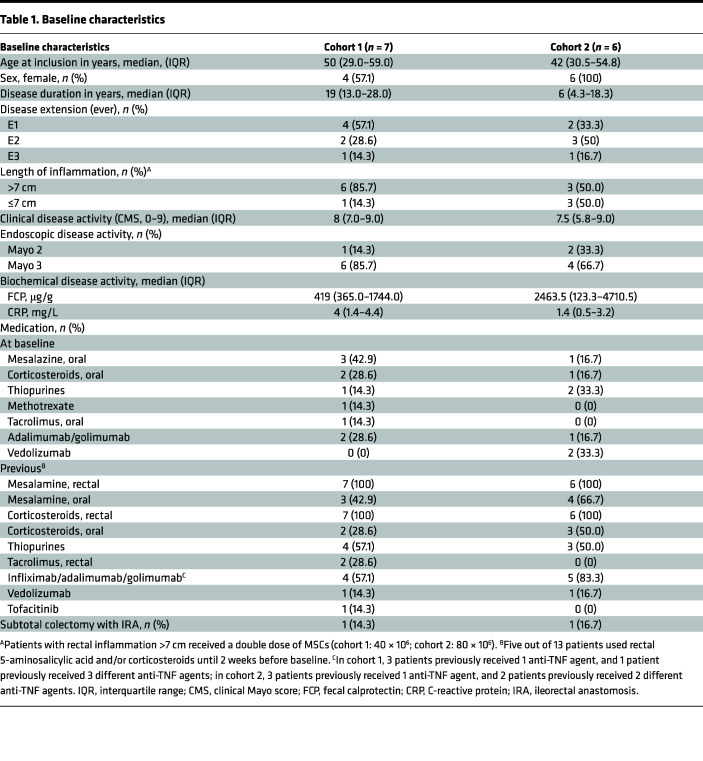
Baseline characteristics

## Version 1 03/23/2023

In-Press Preview

## Version 2 05/08/2023

Electronic publication
