# Supplementary material for: Local administration of mesenchymal stromal cells is safe and modulates the immune compartment in ulcerative proctitis
Source: JCI Insight. 2023 May 8;8(9):e167402. doi: 10.1172/jci.insight.167402 (PMC10243829; doi:10.1172/jci.insight.167402)
Supplement: Supplemental data [file jciinsight-8-167402-s026.pdf]

Supplemental Methods

Mesenchymal stem cell isolation, expansion, and preparation

The MSCs were prepared and expanded as previously described(1). After informed consent was obtained, MSCs were prepared from bone marrow aspirates of three different healthy donors, in accordance with EU Directive 2004/23/EC. Bone marrow was collected at the Leiden University Medical Center (LUMC) by aspiration from the iliac crest under local anesthesia and transferred to Sanquin for isolation and expansion. Mononuclear cells (MNC) were subsequently isolated by COBE2991 separation techniques. Cells were washed and resuspended in MSC culture medium (Dulbecco's modified Eagle's medium – low glucose/Penicillin (100U/mL)/Streptomycin (100U/mL)/10% Fetal Bovine Serum and 4×10^3 cells/ cm^2 were plated in CellStacks. Every 3-4 days, cultures were microscopically examined and medium was refreshed. Cells were passaged, using TrypleLE Select, when >70% confluence was reached. The cells were further expanded through passage P2 in 7-8 days and subsequently cryopreserved at a final concentration of $2x\ 10^6$ viable MSCs/mL in 2% (w/v) human albumin in 0.9% NaCl) with 10% (v/v) DMSO. Then, MSCs were transported to the LUMC, thawed, and cultured through passage 3 to generate freshly harvested cells. The final drug product met the following criteria: >95% of the cells expressed $CD73^+$,$CD90^+$,$CD105^+$, <1% of the cells expressed $CD45^+$, <0.01% of the cells expressed $CD3^+$. Prior to administration, the MSC drug product was packaged in Luer lock syringes in proportions of 10×10^6 viable cells/mL in 0.9% NaCl/2% human albumin solution. The Luer lock syringes were transported on cooling packs to the site of administration. Each primary container contained 10×10^6 viable MSCs per mL and 2-4 mL per syringe in total.

Full inclusion and exclusion criteria

Adults aged 18 years or older were eligible for inclusion. Patients should have ulcerative colitis (UC) confirmed by endoscopic and histologic evidence. Inflammation with an endoscopic Mayo score (EMS) of 2 or 3 should be limited to the rectum, confirmed by endoscopy maximum 3 months before

baseline. Slight inflammation in other parts of the colon was accepted with a maximum EMS of 1. Patients with UP were refractory to conventional medical therapy, which means that at some point during the course of the disease, patients had received rectal 5-ASA therapy and rectal corticosteroid therapy for at least four weeks without an adequate response to treatment. If patients were on rectal therapy, this was stopped 2 weeks before endoscopic MSC therapy and only restarted after 6 weeks. If patients were on oral 5-ASA therapy or corticosteroids, the dose was kept stable for 4 and 2 weeks, respectively prior to study entry and remained the same dose during the first 6 weeks after MSC therapy. Patients treated with 6-mercaptopurine, methotrexate, azathioprine, vedolizumab, or anti-tumor necrosis factor (TNF)-therapy should have been on medication for 3 months with a stable dose for 2 months prior to study entry, and remained on the same dose during the first 6 weeks of MSC therapy. Females of child-bearing age should be non-pregnant, non-breastfeeding, and should use adequate contraception. Patients with expected hospitalization or surgery within 3 months were excluded. Additional exclusion criteria were evidence of any infection needing antibiotic treatment; renal or hepatic failure; use of any investigational drug within one month prior to screening or within five half-lives of the investigational agent (whichever is longer); positive stool culture for enteric pathogens (salmonella, shigella, yersinia, and campylobacter), positive Clostridium difficile toxin A and B, positive stool ova, and parasite exam; tuberculosis or an opportunistic infection within six months prior to screening; positive test for hepatitis B/C, polymerase chain reaction (PCR)-cytomegalovirus, PCR-Epstein-Barr virus, or human immunodeficiency virus serology at baseline. Further exclusion criteria were active malignancy in the past five years; any dysplasia in the colon in the past 5 years; or previous treatment with allogeneic MSCs.

Laboratory methods for supportive research

<u>HLA-antibodies</u>

Detection of HLA-antibodies

Detection of IgG HLA-antibodies in serum samples from baseline and week 6 was performed using Lifecodes Life Screen Deluxe Kit (Immucor) (LMX) and further tested with single bead antigen assay (SBA) Lifecodes LSA Class I and Class II Kits (Immucor) when considered positive for either HLA class I or II. Serum samples from patients with HLA-antibodies at week 6 were directly tested in SBA at week 24. In both assays, antibodies bound to the beads were detected using a PE-conjugated goat-anti-human IgG detection antibody. In the SBA assay, all serum samples were EDTA treated prior to luminex analysis. Samples were acquired on a luminex flow analyzer (LABScan 200) and data were analyzed using the Match IT! Antibody software (version 1.3.1, Lifecodes, Immucor). LMX and SBA results were assigned positive or negative according to the software assignment and the lot-specific cut-off (Match IT! Antibody software).

CDC assay

A complement-dependent cytotoxicity (CDC) assay was performed on 4 patients (#4, #6, #9, and #11), by incubation of patients' serum from baseline and week 6 with a cell panel encompassing all mismatched HLA antigens. After incubation, propidium-iodide was added, in order to stain DNA of lysed lymphocytes and measured with Patimed (Leica).

<u>Fluorescence in situ hybridization</u>

Fluorescence in situ hybridization (FISH) based on X- and Y-chromosome specific probes was performed on formalin-fixed paraffin-embedded biopsies collected 6 weeks after MSC injection, to investigate whether MSCs locally persisted in the tissue mucosa. Since this technique relies on gender differences between patient and MSC-donor, 10/13 biopsies were analysed. One sample was excluded due to technical problems.

Pre-treatment protocol

Firstly, the slides were deparaffinized: 2 times 10 min with Xylol, 5 minutes with ethanol 100%, 5 minutes with ethanol 90%, 5 minutes with ethanol 70%, and then shortly with H_2O. Slides were then immersed in saline Sodium Citric Acid buffer (SSC) for 30 minutes at 85/90 °C and then washed with

H_2O. Then, slides were treated with RNase (100 µg/mL) in PBS for 30 minutes at 37 °C and two times washed for 3 minutes with PBS. After that, a short wash with H_2O. Slides are then immersed for 15 minutes in 0.1% pepsin in 0.01 M HCl at 37 °C, followed by three times a 3-minute wash with PBS. Slides were dehydrated in 70%, 90%, and 100% ethanol, respectively. Slides were air dried. Thereafter, 10 µl probe mix (probe + hybridisation mix) were added to the slides. The probes consisted of a biotin-labeled Y chromosome alpha satellite probe and a digoxigenin-labeled X chromosome alpha satellite probe. The probes were denatured for 8 minutes on a 80 °C hot plate. Slides were hybridized at 37 °C overnight in a humidity chamber with 50% formamide/2xSSC (pH = 7).

Post-hybridization protocol

The first post-hybridization wash twice 5 minutes with 2xSSC/0.1% Tween20 at 37 °C, followed by twice a 5-minute wash with 50% formamide/2xSSC (pH = 7) at 44 °C and once a 5-minute wash 0.1xSSC at 60 °C. Then a short wash with MiliQ and a 3-minute wash with TBST. Incubated for 30 minutes with 1 µl streptavidin Cy-3 and mouse anti-dig FITC in 500 µl TNB at 37 °C. Followed by three times a 3-minute wash with TBST and a short wash with PBS. Slides were dehydrated in respectively, 70%, 90%, and 100% ethanol and air dried. Finally, slides were embedded in DAPI/Citifluor (concentration: 500 ng/mL), 20 µl per slide, coverslip 24x60 mm.

<u>Cytokine analysis</u>

Colon biopsies from baseline and week 6 were analysed by Olink Proteomics (Uppsala, Sweden) using the Explore 384 Inflammation panel. Proteins were extracted from the biopsies by homogenizing in RIPA lysis buffer using 50 mM Tris-HCl pH 7.4, 150 mM NaCl, 1 mM EDTA, 0.1% sodium deoxycholate, 1% Triton X-100 and 1x Complete Protease Inhibitors (Roche # 04693116001) using Tissuelyser LT (Qiagen). Total protein content was determined using a BCA protein assay (ThermoFisher Scientific) and samples were diluted to 0.8 µg/ul for further analysis. Normalized

protein expression (NPX) values, using both internal and external controls for normalization, were compared between baseline and week 6. Five samples were run in duplicates and showed comparable results. 15% (54/369) of the analyzed proteins were not detected in our samples. One sample did not pass quality control (#9, t=0) and was removed from further analysis, together with the paired sample (#9, t=6). This sample also showed lower total protein concentrations compared to the other samples.

<u>Mass cytometry</u>

Isolation of cells from intestinal samples

Cells from intestinal biopsies were collected and isolated as previously described(2, 3). Briefly, intestinal biopsies (n=74) were collected in HBSS (Sigma-Aldrich) medium after endoscopy. Intraepithelial lymphocytes were isolated by treatment with 5 mL HBSS (Sigma-Aldrich) containing 1 mM EDTA (Merck) under rotation for 2 x 45 minutes at 37°C. Next, single cells from the lamina propria (LP) were obtained by enzymatic treatment with a 5 mL 'enzyme-mix' containing IMDM culture medium (Lonza) with 20% FCS, 1 000 U/mL collagenase IV (Worthington), and 10 mg/mL DNase I grade II (Roche Diagnostics) under rotation for 1.5 hours at 37°C. IELs and LP cells were resuspended, filtered through a 70 µm nylon cell strainer, and washed with 0.5% FCS/DPBS (Dulbecco's Phosphate Buffered Saline) (Gibco, Thermofisher). Cells were counted, and a maximum of 2 million cells were resuspended in 1 mL staining buffer (SB) (Fluidigm).

Mass cytometry antibody staining and acquisition

The validated mass cytometry antibody panel is listed in Supplemental Table 10(2). Pre-conjugated metal-labeled antibodies were purchased from Fluidigm (San Francisco, Calfifornia, USA) . The rest of the antibodies were conjugated in-house using the MaxPar X8 antibody labeling kit (Fliudigm Sciences) according to the manufacturer's instructions. After single-cell isolation, cells were washed with SB (Fluidigm) and incubated with 1 mL 2000 mM rhodium DNA intercalator diluted in SB (1:2000) for 15 minutes to stain dead cells. Then, cells were washed with SB and incubated for 10

minutes with Fc Receptor blocking solution (Biolegend). Next, cells were stained with metal-conjugated antibodies (as listed in Supplemental Table 10) for 45 min at room temperature. After staining, cells were washed three times with SB and incubated with 1 mL 500 mM iridium DNA intercalator (Fluidigm) diluted in MaxPar Fix and Perm Buffer (Fluidigm) (1:4000) at 4°C overnight, or up to 48 hours, to discriminate single-cells. Cells were washed three times with SB. Before data acquisition, cells were diluted in distilled water containing 1:10 diluted EQ Four Element Calibration Beads (Fluidigm Sciences). Samples were acquired at the Helios mass cytometer (Fluidigm, San Francisco, CA, USA) with the narrow-bore injector at the Flow Core Facility (FCF) of the Leiden University Medical Center. CyTOF data were acquired and analyzed on-the-fly, using dual-count mode and noise-reduction on. All other settings were either default settings or optimized with tuning solution, as instructed by Fluidigm. After data acquisition, the mass bead signal was used to normalize the short-term signal fluctuations with the reference EQ passport P13H2302 during the course of each experiment. Samples from the same patient were processed and acquired on the same day.

Mass cytometry data analysis

First, normalized FCS files were checked for quality control of the staining and pre-gated for single live CD45$^+$ cells, removing duplicates, beads, dead cells, and debris in the FlowJo software V10.8.1. A reference PBMC sample was included in each experiment to account for technical variation. All reference PBMCs were obtained from blood from the same healthy individual. ComBat was applied to align the PBMC reference samples and corresponding patient samples to correct for batch effects(4). The markers CD66b, CD15, Nkp44, Nkp46, c-kit, CD40, CD80, and PD-L1 were not present in the reference PBMCs at sufficient levels to scale and were thus not normalized. For further analysis, these antibodies were not used for clustering but were shown for marker expression overlays and interpretation of data. CD45$^+$ cells were sample-tagged, hyperbolic ArcSinh transformed with a cofactor of 5, and subjected to dimensionality reduction analysis in

Cytosplore(5). First, major immune lineages (Figure 4A) were identified at the overview level of a 5-level hierarchical stochastic neighbor embedding (HSNE) analysis on $CD45^+$ cells from control and case rectal biopsies, before and after 6 weeks of MSC therapy (3.4 million cells) with default perplexity and iterations (30 and 1000, respectively)(6, 7). This global overview contained clusters of myeloid cells ($CD66b^+$ granulocytes and $CD66b^-$ mononuclear phagocytes), $CD4^+$ T cells, $CD8^+$ T cells, double-negative (DN) T cells, γδ T cells, innate lymphoid cells (ILCs), B cells, and $CD45^+Lineage^-$ cells. $CD66b^+$ granulocytes and $CD66b^-$ myeloid cells were identified within the myeloid cell compartment at the overview level of a 3-level HSNE analysis on 1.8 million cells. $CD66b^-$ myeloid cells were further analyzed using tSNE. The other major immune lineage clusters were analyzed separately in a data-driven manner up to a maximum number of 0.5×10^6 landmarks using t-distributed stochastic neighbor embedding (tSNE)(7) with a default perplexity (30) and iterations (1000). The cells from each lineage were clustered using a sigma value of 15. Clustering of the data was performed by Gaussian mean shift (GMS) clustering in Cytosplore, and clusters that showed high similarity in ArcSinh5-transformed median expression of all markers were merged. Quantification of frequencies of clusters in each sample was performed in GraphPad Prism v9.

References

1. Molendijk I et al. Allogeneic Bone Marrow - Derived Mesenchymal Stromal Cells Promote Healing of Refractory Perianal Fistulas in Patients With Crohn's Disease. *Gastroenterology* 2015;149(4):918-927.e6.

2. van Unen V et al. Identification of a Disease-Associated Network of Intestinal Immune Cells in Treatment-Naive Inflammatory Bowel Disease. *Front Immunol* 2022;13. doi:10.3389/fimmu.2022.893803

3. van Unen V et al. Mass Cytometry of the Human Mucosal Immune System Identifies Tissue- and Disease-Associated Immune Subsets. *Immunity* 2016;44(5):1227–1239.

4. Johnson WE, Li C, Rabinovic A. Adjusting batch effects in microarray expression data using empirical Bayes methods. *Biostatistics* 2007;8(1):118–127.

5. Höllt T et al. Cytosplore: Interactive Immune Cell Phenotyping for Large Single-Cell Datasets. *Computer Graphics Forum* 2016;35(3):171–180.

6. Ma K-L, Santucci G, van Wijk JJ. *Hierarchical Stochastic Neighbor Embedding*. 2016:

7. van Unen V et al. Visual analysis of mass cytometry data by hierarchical stochastic neighbour embedding reveals rare cell types. *Nat Commun* 2017;8(1). doi:10.1038/s41467-017-01689-9

Supplemental Tables and Figures

Supplemental Table 1. Adverse events reported in MSC-treated patients

**Adverse events**	*Baseline - week 6 (n)*	*week 6 - week 24 (n)*	*week 24 - week 52 (n)*
abdominal pain	6	4	6
abdominal cramps	4	5	3
nausea	4	3	2
diarrhea	2	0	1
vomiting	3	0	0
fecal urge	2	1	4
flatulence	3	2	1
obstipation	1	0	2
Appendicitis	0	0	1
bloating	0	1	1
loss of appetite	0	1	0
general malaise	0	1	0
cold symptoms	2	2	0
fever (≥ 38.5 °C)	2	1	0
dyspnoea	0	1	0
palpitations	0	1	0
profuse sweating	0	1	0
chest pain	0	1	0
dry cough	0	1	0
swollen glands	1	0	0
sore throat	1	0	0
head ache	1	3	2
Fatigue	2	2	2
dizziness	0	1	0
arthralgia	1	3	1
urinary tract infection	0	1	0

Supplemental Table 2. Full Mayo score, Clinical Mayo score, Endoscopic Mayo score and Rectal Bleeding score

Patient	Cohort	Full Mayo score (0-12)				Clinical Mayo score (0-9)				Endoscopic Mayo score (0-3)				Rectal bleeding score (0-3)			
		Baseline	*Week 2*	*Week 6*	*Week 24*	*Baseline*	*Week 2*	*Week 6*	*Week 24*	*Baseline*	*Week 2*	*Week 6*	*Week 24*	*Baseline*	*Week 2*	*Week 6*	*Week 24*
1	1	8	9	7	1	5	6	4	0	3	3	3	1	0	1	1	0
2	1	12	11	12	7	9	8	9	5	3	3	3	2	3	2	3	0
3	1	11	10	5	3	8	7	3	2	3	3	2	1	3	3	1	1
4	1	10	8	6	7	8	7	5	5	2	1	1	2	3	2	1	1
5	1	10	8	8	6	7	5	5	4	3	3	3	2	2	1	1	1
6	1	12	11	10	1	9	8	7	0	3	3	3	1	3	3	2	0
7	1	12	12	12	4	9	9	9	3	3	3	3	1	3	3	3	2
8	2	7	2	6	1	5	1	4	1	2	1	2	0	3	1	3	0
9	2	12	11	10	7	9	8	7	4	3	3	3	3	3	2	1	0
10	2	10	8	8	9	7	5	5	6	3	3	3	3	3	1	1	1
11	2	11	9	9	4	8	6	7	3	3	3	2	1	3	2	2	1
12	2	9	6	6	4	6	4	4	2	3	2	2	2	0	0	0	0
13	2	11	10	5	2	9	8	3	2	2	2	2	0	3	3	1	0

The full Mayo score includes the following subscores: rectal bleeding score, stool frequency, physician's global assessment, and endoscopic mayo score. The clinical Mayo score is the full Mayo score without the endoscopic Mayo score.

Supplemental Table 3. Overview of medication use

Patient	Cohort	Prior medical therapies	Full Mayo score	Therapies[a]	Full Mayo score	Changes in therapies	Changes in therapies	Full Mayo score	Changes in therapies
			baseline		*week 6*	*week 6 - week 12*	*week 12 - 24*	*week 24*	*week 24 - 52*
1	1	5-ASA (R, O); CS (R)	8	5-ASA (O)	7	start: TIO; 5-ASA (R); CS (R)	stop: CS (R)	1	
2	1	5-ASA (R); CS (R); TIO; MTX; ADA	12	MTX; ADA	12		stop: MTX; ADA<br>start: VEDO	7	
3	1	5-ASA (R, O); CS (R)	11	5-ASA (O)	5		start: 5-ASA (R)	3	start: IFX; TIO
4	1	5-ASA (R, O); CS (R, O); TAC (R); IFX; TOFA	10	CS (O)	6		start: USTE	7	start: 5-ASA (R)
5	1	5-ASA (R, O); CS (R, O); TIO; TAC (O); IFX; ADA; GOL; VEDO	10	TIO; TAC (O); GOL	8	GOL higher dose		6	stop: GOL<br>start: CS (R); TOFA
6	1	5-ASA (R, O); CS (R, O); TAC (R)	12	-	10	start: 5-ASA (R); CS (R); TAC (R)		1	
7	1	5-ASA (R, O); CS (R, O); TIO; IFX	12	5-ASA (O); CS (O)	12		start: 5-ASA (R); CS (R); USTE	4	
8	2	5-ASA (R, O); CS (R, O)	7	-	6	start: 5-ASA (O); CS (R)		1	
9	2	5-ASA (R, O); CS (R, O)	12	5-ASA (O); CS (O); VEDO	10		stop: VEDO<br>start: 5-ASA (R); USTE	7	
10	2	5-ASA (R, O); CS (R); TIO	10	TIO; VEDO	8			9	stop: VEDO<br>start: 5-ASA (R); CS (R); IFX<br>switch: USTE*
11	2	5-ASA (R, O); CS (R, O); TIO; TAC (R); IFX; VEDO	11	VEDO	9	start: 5-ASA (R)		4	start: CS (R)
12	2	5-ASA (R, O); CS (R); TIO; IFX; GOL; VEDO	9	TIO	6	start: 5-ASA (R)		4	start: CS (R); 5-ASA (R); USTE
13	2	5-ASA (R, O); CS (R, O); TIO; IFX; ADA	11	ADA	5		stop ADA<br>start: USTE	2	

Full Mayo score includes stool frequency, rectal bleeding, endoscopic findings, and physician's global assessment. The indicated thiopurines include azathioprine, 6-mercaptopurine, and tioguanine.
[a] Patient 4, 7 and 9 used oral corticosteroids (budesonide 9 mg) at baseline. Patient 4 tapered with a dose of 9 mg every other day from week 8, patient 7 continued with a dose of 9 mg up to week 10 and patient 9 tapered with a dose of 9 mg every other day from week 6.
R: rectal; O: oral; 5-ASA = 5-amino salicylic acid; CS: corticosteroids; TIO: thiopurines; MTX = methotrexate; ADA = adalimumab; VEDO: vedolizumab; IFX: infliximab; TAC = tacrolimus; TOFA: tofacitinib; USTE = ustekinumab; GOL: golimumab.
* This patient first used infliximab and switched after 6 months to ustekinumab.

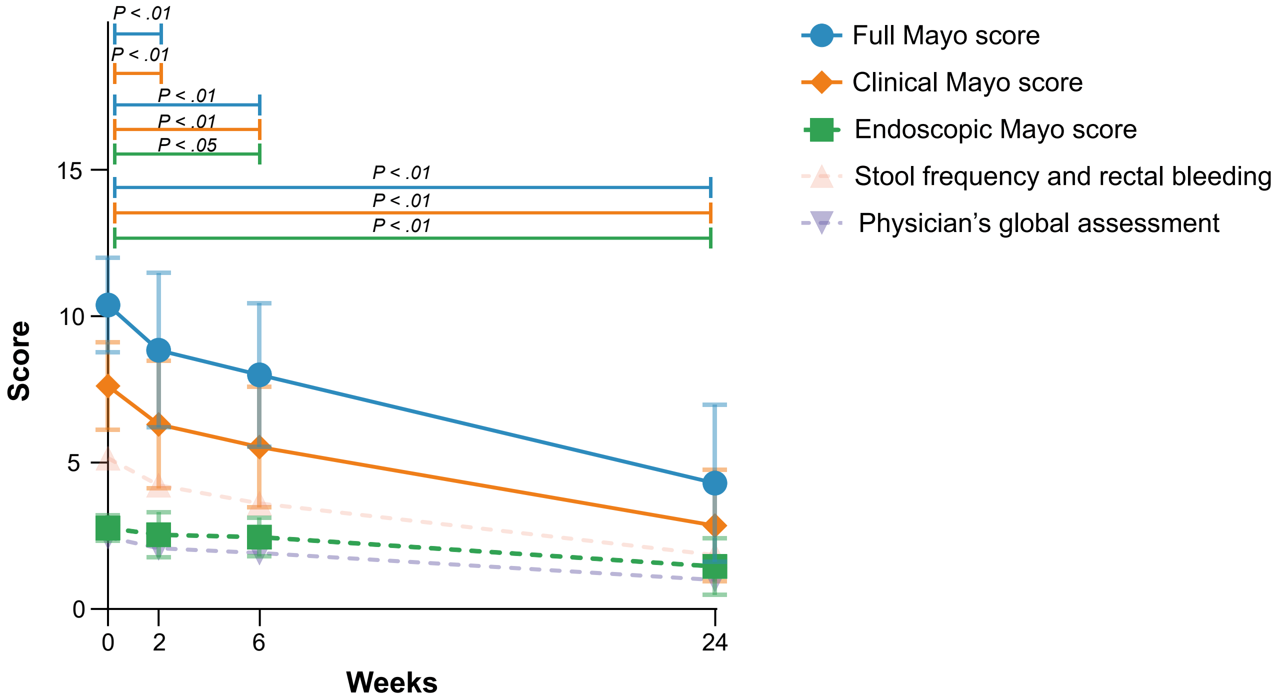

Supplemental Figure 1. Mean full Mayo score with corresponding sub-scores at baseline and follow-up visits (weeks 2, 6, and 24). Dots represent the mean with standard deviations. Wilcoxon signed-rank test is performed.

Supplemental Table 4. Fecal calprotectin and C-reactive protein values

Patient	Cohort	Fecal Calprotectin (ug/g)											C-reactive protein (mg/L)				
		Baseline	*Week 2*	*Week 6*	*Week 12*	*Week 24*	*Δ2*	*Δ6*	*Δ12*	*Δ24*	*Baseline – week 2 decrease of > 150 ug/g*	*Baseline – week 6 decrease of > 150 ug/g*	*Baseline*	*Week 2*	*Week 6*	*Week 12*	*Week 24*
1	1	378	678	1606	59	7.2	79%	325%	-84%	-98%	No	No	4.4	6.9	6	36.6	1.3
2	1	451	373	854	567	96	-17%	89%	26%	-79%	No	No	1.4	2	1.3	1.4	1.2
3	1	419	293	191	475	357	-30%	-54%	13%	-15%	No	No	0.5	0.8	0.4	2.7	1.2
4	1	365	163	40	1202	85	-55%	-89%	229%	-77%	Yes	Yes	4.1	1.2	2.4	0.6	2.6
5	1	1744	63	364	15	188	-96%	-79%	-99%	-89%	Yes	Yes	55.1	6.2	2.6	4.8	4.7
6	1	3270	1089	291	106	35	-67%	-91%	-97%	-99%	Yes	Yes	4	2.1	2.3	1.7	1.6
7	1	216	1830	1023	2095	2788	747%	374%	870%	1191%	No	No	3.2	4.2	13.3	6.6	4.7
8	2	3341	77	2161	6.1	27	-98%	-35%	-100%	-99%	Yes	Yes	0.7	1.5	1.3	1.2	2.6
9	2	1586	1358	2795	3479	939	-14%	76%	119%	-41%	Yes	No	3.1	7	19.2	36.4	7.9
10	2	5462	555	35330	4720	4922	-90%	547%	-14%	-10%	Yes	No	3.6	2.9	2.3	2.3	2.7
11	2	4460	10400	7470	1624	8193	133%	67%	-64%	84%	No	No	2	1.7	3	2.1	5.7
12	2	137	134	449	11	17	-2%	228%	-92%	-88%	No	No	0.7	0.4	0	0	0.4
13	2	82	157	38	42	13	91%	-54%	-49%	-84%	No	No	0	0	0	0	0

Δ2, 6, 12, 24 = the percentage of increased or decreased fecal calprotectin values at week 2, 6, 12, or 24 compared to baseline.

Supplemental Table 5. Histologic activity in Geboes score

**Patient**	**Cohort**	*Baseline*	*Week 6*	*Week 24*
1	1	>12	>12	≤ 6
2	1	>12	>12	≤ 6
3	1	>12	7-12	>12
4	1	≤ 6	≤ 6	≤ 6
5	1	>12	7-12	7-12
6	1	>12	7-12	≤ 6
7	1	7-12	7-12	≤ 6
8	2	≤ 6	≤ 6	≤ 6
9	2	>12	≤ 6	>12
10	2	>12	>12	>12
11	2	>12	7-12	7-12
12	2	≤ 6	≤ 6	≤ 6
13	2	7-12	≤ 6	≤ 6

The continuous scale (ranging from 0-22) of the Geboes score was used and biopsies were evaluated at week 6 and 24 compared to baseline, according to three categories: histological remission (GS ≤6), histological response (GS 7-12), and no response (GS >12).

Supplemental Table 6. Questionnaires regarding the quality of life and patient-reported outcome measures

Questionnaires	*Baseline*	*Week 2*		*Week 6*		*Week 12*		*Week 24*	
			p-value[a]		*p-value*[a]		*p-value*[a]		*p-value*[a]
**sIBDQ**	41 (34-49.5)	45 (37.5-52)	*0.123*	47 (42.5-55)	*0.021*	59 (39.5-62)	*0.001*	56 (44.5-63.5)	*0.001*
**SF-36**									
- PF	24 (17.5-28.5)	23 (18.5-29)	*0.095*	26 (21.5-29)	*0.012*	25 (19.5-29.5)	*0.084*	25 (21.5-30)	*0.037*
- RP	8 (5-12)	10 (5-18)	*0.097*	10 (6.5-15.5)	*0.020*	14 (8.5-19.5)	*0.008*	14 (8.5-20)	*0.002*
- RE	12 (8-15)	11 (9-15)	*0.569*	12 (9.5-15)	*0.234*	15 (8.5-15)	*0.261*	12 (8-15)	*0.373*
- MH	20 (16.5-21.5)	21 (17-23)	*0.132*	20 (16-22.5)	*0.404*	22 (18-23.5)	*0.057*	21 (17.5-23)	*0.073*
- VT	9 (7-12)	10 (7.5-14)	*0.301*	9 (8.5-12)	*0.126*	11 (10-13.5)	*0.068*	13 (10-15)	*0.012*
- PN	33 (27.5-52)	39 (27.5-60)	*0.123*	44 (33-54.5)	*0.037*	22 (30-55)	*0.171*	44 (33-57.5)	*0.064*
- GH	11 (9.5-15)	11 (9-14.5)	*0.944*	12 (9-17)	*0.251*	12 (10-18)	*0.031*	13 (10.5-20.5)	*0.016*
- SF	6 (3.5-8.5)	7 (4-9)	*0.429*	7 (5.5-8.5)	*0.085*	8 (4.5 - 10)	*0.011*	8 (5.5 - 10)	*0.012*
- HC	2 (1.0-3)	2 (1.5-3)	*0.593*	3 (2-4)	*0.076*	3 (2.5-3.5)	*0.026*	4 (3-4)	*0.007*
**mHealth**	30 (22.5-31.5)	24 (16.5-31.5)	*0.116*	16 (11-26.5)	*0.004*	15 (8-27)	*0.003*	13 (3-18.5)	*0.001*

[a] The p-value corresponding to the Wilcoxon signed rank test that is performed to compare week 2, 6, 12, and 24 to baseline, respectively.
sIBDQ = short Inflammatory Bowel Disease Questionnaire; SF-36 = Short Form-36; PF = physical functioning; RP = role physical; RE = role emotional; MH = mental health; VT = vitality; PN = physical pain; GH = general health; SF = social functioning; HC = health change; mHealth = mobile health. Values represented in median (interquartile range).

Supplemental Table 7. MSC persistence and DSAs, and response

Patient				Response			
	DSAs present	*Positive CDC assay*	*MSC persistence in tissue (FISH)*	*Clinical (FMS)*	*Endoscopic (EMS)*	*Biochemical (FCP)*	*Histological (GS)*
1	no	-	-	no	no	no	no
3	no	-	-	response	response	improvement	response
12	no	-	-	no	response	no	remission
13	no	-	-	yes	no	improvement	remission
7	no	-	no	no	no	no	response
8	no	-	no	no	no	improvement	remission
11	yes	yes	no	no	response	no	response
6	yes	no	no	no	no	improvement	response
9	yes	no	no	no	no	no	remission
4	yes	no	yes	response	response	improvement	remission
2	no	no	yes	no	no	no	no
5	no	no	yes	no	no	improvement	response
10	no	no	yes	no	no	no	no

DSAs = donor-specific antibodies; CDC = complement-dependent cytotoxicity; FISH = fluorescent in situ hybridization; FMS = full Mayo score; EMS = endoscopic Mayo score; FCP = feces calprotectin; GS = Geboes score.

Supplemental Table 8. Presence of class I and II DSAs after 6 weeks induced by allogeneic bm-MSC therapy

**Patient #4**	**HLA-A***		**HLA-B***		**HLA-C***		**HLA-DRB1***		**HLA-DRB 3-4-5***		**HLA-DQB1***		**HLA-DPB1***	
HLA-typing patient	01:01:01		08:01:01		07:01:01		01:01:01	03:01:01	3 (01:01:02)		02:01:01	05:01:01	04:01:01	
HLA-typing MSC-donor #2	02:01:01	68:01:02	40:02:01	44:27:01	02:02:02	07:04:01	11:01:01	16:01:01	3	5	03:01:01	05:02:01	04:01:01	15:01:01
SA Luminex class I														
- *baseline*	-													
- *week 6*	**A2**, **A68**, **B44**, **B61**													
- *week 24*	-													
SA Luminex class II														
- *baseline*	-													
- *week 6*	-													
- *week 24*	-													
**Patient #6**	**HLA-A***		**HLA-B***		**HLA-C***		**HLA-DRB1***		**HLA-DRB 3-4-5***		**HLA-DQB1***		**HLA-DPB1***	
HLA-typing patient	01:01:01	30:01:01	08:01:01	38:01:01	07:01:01	12:03:01	13:01:01		3 (01:01:02)		06:03:01		02:01:02	04:01:01
HLA-typing MSC-donor #3	02:01:01	11:01:01:01	15:01:01	35:01:01	04:01:01		01:03	04:04:01	4		03:02/03:11	05:01/05:51	04:01:01	
SA Luminex class I														
- *baseline*	-													
- *week 6*	**A2**, **B62**, **B35**													
- *week 24*	-													
SA Luminex class II														
- *baseline*	-													
- *week 6*	-													
- *week 24*	-													

Supplemental Table 8 continued. Presence of class I and II DSAs after 6 weeks induced by allogeneic bm-MSC therapy

**Patient #9**	**HLA-A***		**HLA-B***		**HLA-C***		**HLA-DRB1***		**HLA-DRB 3-4-5***		**HLA-DQB1***		**HLA-DPB1***	
HLA-typing patient	02:01:01	11:01:01	57:01:01		06:02:01		07:01:01	13:02:02	3 (01:01:02)	4 (01:02:02)	03:03:02	06:04:01	03:01:01	13:01:01
HLA-typing MSC-donor #3	02:01:01	11:01:01	15:01:01	35:01:01	04:01:01		01:03	04:04:01	4		03:02/03:11	05:01/05:51	04:01:01	
SA Luminex class I														
- *baseline*	-													
- *week 6*	**B62**, **B35**													
- *week 24*	-													
SA Luminex class II														
- *baseline*	-													
- *week 6*	**DR1**													
- *week 24*	-													
**Patient #11**	**HLA-A***		**HLA-B***		**HLA-C***		**HLA-DRB1***		**HLA-DRB 3-4-5***		**HLA-DQB1***		**HLA-DPB1***	
HLA-typing patient	11:01:01	30:01:01	13:02:01	35:01:01	04:01:01	06:02:01	01:01:01	07:01:01	4		02:02:01	05:01:01	04:01:01	17:01:01
HLA-typing MSC-donor #3	02:01:01	11:01:01:01	15:01:01	35:01:01	04:01:01		01:03	04:04:01	4		03:02/03:11	05:01/05:51	04:01:01	
SA Luminex class I														
- *baseline*	**A2**													
- *week 6*	**A2**, A23, A24, A2403, A68, A69, B57, B58													
- *week 24*	**A2**, A24, A68, A69, B57, B58													
SA Luminex class II														
- *baseline*	**DR4**, **DQ3/7**, **DQ3/8**, **DQ3/9**													
- *week 6*	**DR4**, **DQ3/7**, **DQ3/8**, **DQ3/9**													
- *week 24*	**DR4**, **DQ3/7**, **DQ3/8**, **DQ3/9**													

In **red** the donor-specific HLA antibodies detected in the patients' sera. In red the HLA-antibodies that occur due to epitope spreading. B40 is composed of B60 and B61 split antigen serotypes. B62 is the largest of many split antigens of the broad antigen B15.
HLA = human leukocyte antigen; MSC = mesenchymal stromal cell; SA = single antigen.

Supplemental Table 9. Mean fluorescence intensity values of anti-HLA antibodies

Patient #4				Patient #6				Patient #9				Patient #11			
Allele, MFI	*Baseline*	*Week 6*	*Week 24*	Allele, MFI	*Baseline*	*Week 6*	*Week 24*	Allele, MFI	*Baseline*	*Week 6*	*Week 24*	Allele, MFI	*Baseline*	*Week 6*	*Week 24*
**A2**	**-**	**4176**	**-**	**A2**	**-**	**944**	**-**	**B62**	**-**	**4908**	**-**	**A2**	**1777**	**19825**	**12147**
**A68**	**-**	**3859**	**-**	**B62**	**-**	**2637**	**-**	**B35**	**-**	**3472**	**-**	A23	-	2692	-
**B44**	**-**	**1910**	**-**	**B35**	**-**	**1636**	**-**	**DR1**	**-**	**4728**	**-**	A24	-	5811	1762
**B61**	**-**	**1615**	**-**									A2403	-	6186	1997
												A68	1035	18240	11153
												A69	954	16153	9252
												B57	-	11692	1990
												B58	-	10001	1286
												**DR4**	**2445**	**6282**	**4912**
												**DQ3/7**	**1887**	**2736**	**3472**
												**DQ3/8**	**2037**	**3150**	**3830**
												**DQ3/9**	**1806**	**2705**	**3593**

In **red** the donor-specific HLA antibodies detected in the patients' sera. In red (underlined) the HLA-antibodies that occur due to epitope spreading.
MFI = mean fluorescence intensity.

Supplemental Table 10. CDC assay

**Patient #9**	*Baseline*	*Week 6*
DSAs to be tested: B62, B35, DR1		
HLA-typing cell type I (spleen):<br>A2, A11, B44, B12, B57, B17, Bw4, Cw5, Cw6,<br>**DR1** , DQ5, DQ1		
CDC	neg	neg
CDC+DTT	neg	neg
HLA-typing cell type II (PBMC):<br>A1, A2, **B62**, B15, B57, B17, Bw4, Bw6, Cw9, Cw3, Cw6<br>DR13, DR6, DR7, DR52, DQ6, DQ1, DQ9, DQ3		
CDC	neg	neg
CDC+DTT	neg	neg
**Patient #11**	*Baseline*	*Week 6*
DSAs to be tested: A2, DR4, DQ3		
HLA-typing cell type I (PBMC):<br>**A2**, A11, B35, Bw6, Cw4<br>DR103, **DR4**, DR53, DQ5, **DQ8, DQ3**		
CDC	neg	pos
CDC+DTT	neg	pos
Negative: score 0-1; positive: score 2-5. From score 2 there is 27% more cell death compared to score 0.<br>DSAs = donor-specific antibodies; HLA = human leukocyte antigen; CDC = complement-dependent cytotoxicity;<br>DTT = dithiothreitol; neg = negative; pos = positive; PBMC = peripheral blood mononuclear cells		

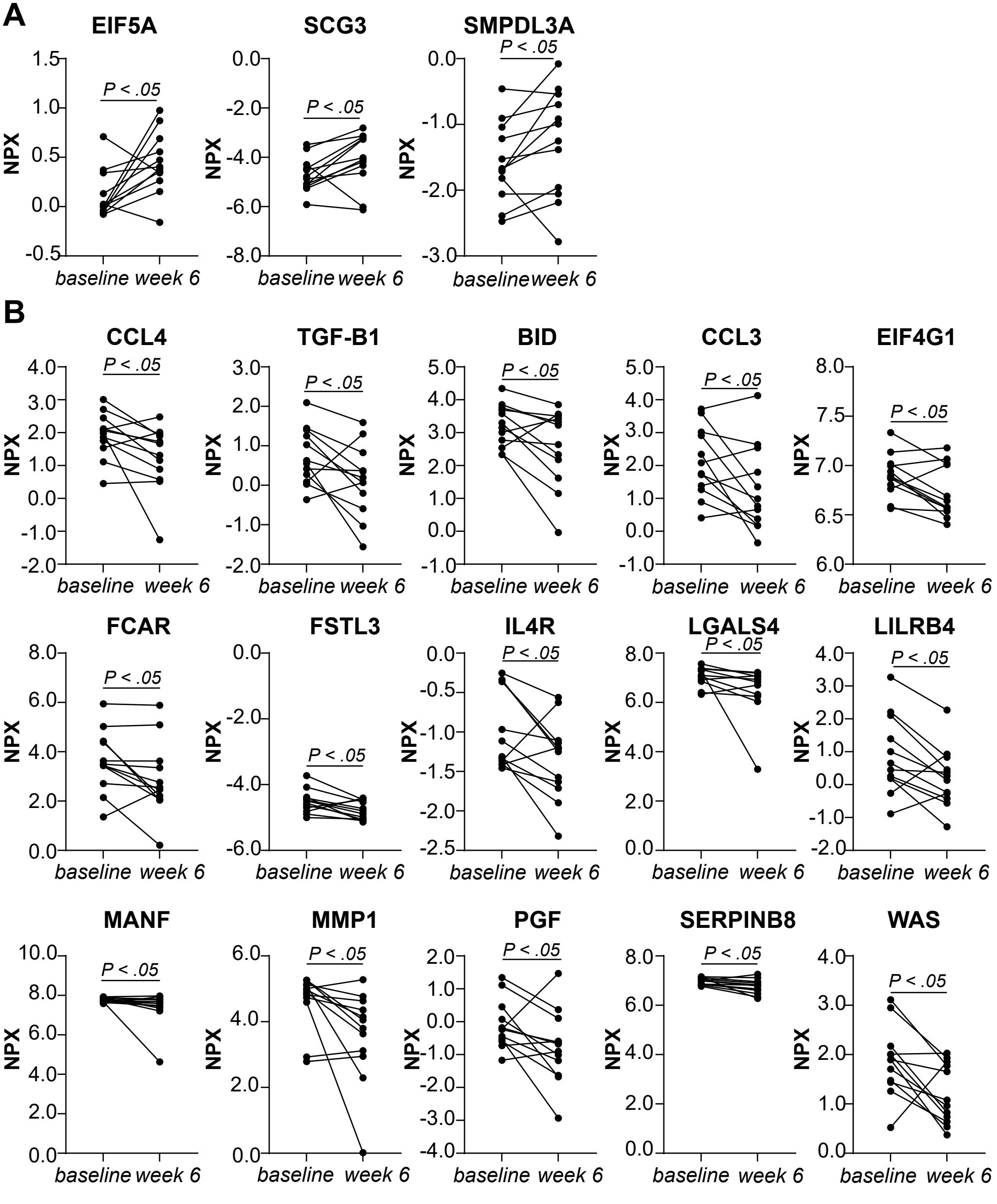

Supplemental Figure 2. Olink proteomic analysis showing modulation of inflammatory proteins upon MSC therapy (**A**) Proteins that showed increased levels 6 weeks after MSC therapy compared to baseline. (**B**) Proteins that were downregulated 6 weeks after MSC therapy compared to baseline. Wilcoxon signed-rank test was performed on 12 paired samples from case biopsies at baseline and week 6.

Supplemental Table 11. Mass cytometry antibody panel

	Antigen	Tag	Clone	Supplier	Cat#	Final dilution
1	CD8a	^{146}Nd	RPA-T8	FLM	3146001B	1/100
2	CD11c	^{162}Dy	Bu15	FLM	3162005B	1/200
3	CD127	^{165}Ho	AO19D5	FLM	3165008B	1/200
4	CD38	^{172}Yb	HIT2	FLM	3172007B	1/200
5	CD69	^{144}Nd	FN50	FLM	3144018B	1/100
6	CD11b	^{209}Bi	ICRF44	FLM	3209003B	1/100
7	CD45	^{89}Y	HI30	FLM	3089003B	1/100
8	CCR6	^{141}Pr	G034E3	FLM	3141003A	1/100
9	C-Kit	^{143}Nd	104D2	FLM	3143001B	1/100
10	CD4	^{145}Nd	RPA-T4	FLM	3145001B	1/100
11	CD16	^{148}Nd	3G8	FLM	3148004B	1/100
12	CD25	^{149}Sm	2A3	FLM	3149010B	1/100
13	CD123	^{151}Eu	6H6	FLM	3151001B	1/100
14	CD7	^{153}Eu	CD7-6B7	FLM	3153014B	1/100
15	TIGIT	^{154}Sm	MBSA4	FLM	3154016B	1/100
16	CCR7	^{159}Tb	G043H7	FLM	3159003A	1/100
17	CD161	^{164}Dy	HP-3G10	FLM	3164009B	1/100
18	CD27	^{167}Er	O323	FLM	3167002B	1/100
19	CD45RA	^{169}Tm	HI100	FLM	3169008B	1/100
20	CD3	^{170}Er	UCHT1	FLM	3170001B	1/100
21	PD-1	^{175}Lu	EH 12.2H7	FLM	3175008B	1/100
22	CD56	^{176}Yb	NCAM16.2	FLM	3176008B	1/100
23	TCRgd	^{152}Sm	11F2	FLM	3152008B	1/50
24	CD40	^{142}Nd	5C3	FLM	3142010B	1/100
25	PD-L1	^{156}Gd	29E.2A3	FLM	3156026B	1/200
26	CD80	^{161}Dy	2D10.4	FLM	3161023B	1/100
27	CD15	^{115}In	W6D3	BioL	323035	1/50
28	CD5	^{160}Gd	UCHT2	BioL	300627	1/50
29	HLA-DR	^{168}Er	L243	BioL	307651	1/300
30	IgM	^{150}Nd	MHM88	BioL	314527	1/100
31	CD103	^{155}Gd	Ber-ACT8	BioL	350202	1/100
32	CD20	^{163}Dy	2H7	BioL	302343	1/200
33	CD28	^{171}Yb	CD28.2	BioL	302937	1/100
34	CD45RO	^{173}Yb	UCHL1	BioL	304239	1/100
35	CD122	^{158}Gd	TU27	BioL	339015	1/50
36	CD8b	^{166}Er	SIDI8BEE	ebio	15257407	1/50
37	NKp46	^{174}Yb	9E 2	BioL	331902	1/40
38	Nkp44	^{147}Sm	253415	R&D systems	MAB22491	1/40
39	CD14	Qdot800	TüK4	Invitrogen/ThermoFisher	Q10064	1/1000
40	CD57	^{194}Pt	HCD57	BioL	322325	1/200
41	CD66b	^{198}Pt	6/40C	BioL	392902	1/40
FLM = Fluidigm; BioL = Biolegend; eBio = eBioschience™						

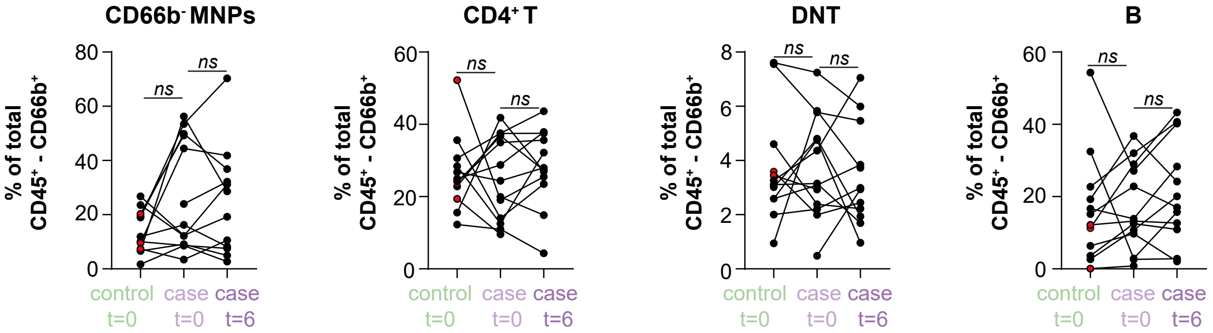

Supplemental Figure 3. High-dimensional analysis of control and case biopsies at baseline and after 6 weeks of local MSC therapy. Frequencies of the CD66b$^-$ mononuclear phagocytes, CD4$^+$ T cells, double-negative T cells and, B cells from individual samples (control (n=12) and case biopsies (n=13) at baseline and case biopsies (n=12) at week 6); Each dot represents an individual sample. Red colored dot indicated mild inflammation (endoscopic Mayo score 1). ns, not significant. Wilcoxon signed-rank test was performed.
